# Transcriptional Regulation of the Glucose-6-Phosphate/Phosphate Translocator 2 Is Related to Carbon Exchange Across the Chloroplast Envelope

**DOI:** 10.3389/fpls.2019.00827

**Published:** 2019-06-27

**Authors:** Sean E. Weise, Tiffany Liu, Kevin L. Childs, Alyssa L. Preiser, Hailey M. Katulski, Christopher Perrin-Porzondek, Thomas D. Sharkey

**Affiliations:** ^1^MSU-DOE Plant Research Laboratory, Michigan State University, East Lansing, MI, United States; ^2^Department of Biochemistry and Molecular Biology, Michigan State University, East Lansing, MI, United States; ^3^Department of Plant Biology, Michigan State University, East Lansing, MI, United States; ^4^Plant Resilience Institute, Michigan State University, East Lansing, MI, United States

**Keywords:** glucose 6-phosphate, G6P, GPT2, RRTF1, TPT, GAP, PGA, redox regulation

## Abstract

The exchange of reduced carbon across the inner chloroplast envelope has a large impact on photosynthesis and growth. Under steady-state conditions it is thought that glucose 6-phosphate (G6P) does not cross the chloroplast membrane. However, growth at high CO_2_, or disruption of starch metabolism can result in the *GPT2* gene for a G6P/P_i_ translocator to be expressed presumably allowing G6P exchange across the chloroplast envelope. We found that after an increase in light, the transcript for GPT2 transiently increases several 100-fold within 2 h in both the Col-0 and WS ecotypes of *Arabidopsis thaliana*. The increase in transcript for GPT2 is preceded by an increase in transcript for many transcription factors including Redox Responsive Transcription Factor 1 (RRTF1). The increase in *GPT2* transcript after exposure to high light is suppressed in a mutant lacking the RRTF1 transcription factor. The *GPT2* response was also suppressed in a mutant with a T-DNA insert in the gene for the triose-phosphate/P_i_ translocator (TPT). However, plants lacking TPT still had a robust rise in *RRTF1* transcript in response to high light. From this, we conclude that both RRTF1 (and possibly other transcription factors) and high amounts of cytosolic triose phosphate are required for induction of the expression of *GPT2*. We hypothesize that transient *GPT2* expression and subsequent translation is adaptive, allowing G6P to move into the chloroplast from the cytosol. The imported G6P can be used for starch synthesis or may flow directly into the Calvin-Benson cycle via an alternative pathway (the G6P shunt), which could be important for regulating and stabilizing photosynthetic electron transport and carbon metabolism.

## Introduction

Plants live in a stochastic environment with drivers of photosynthesis such as CO_2_ availability and light changing rapidly as abiotic factors such as cloud cover or humidity change over the course of minutes and hours. Hexose phosphate transport into plastids has been shown to allow plants achieve higher rates of photosynthesis over the course of several days when irradiance is increased (Dyson et al., 2015). This process of acclimation involves the differential expression (DE) and changes in abundance of hundreds of proteins in the chloroplast (Miller et al., 2017). The glucose-6-phosphate/phosphate translocator 2 (GPT2) in *Arabidopsis* is of particular interest as the expression for this protein increases rapidly, hours as opposed to days, when irradiance is increased (Athanasiou et al., 2010). We wanted to better understand the more rapid changes in gene expression, as opposed to acclimation, that occur when the photosynthetic rate of a plant increases. To this end, we focused on the role of GPT2 in carbon exchange across the chloroplast envelope.

Hexose phosphate transport into plastids was first documented 30 years ago. It was determined that starch synthesis in heterotrophic amyloplasts used imported hexose phosphates rather than triose phosphates (Keeling et al., 1988; Tyson and ap Rees, 1988). In 1995 it was discovered that autotrophic chloroplasts could also transport glucose 6-phosphate (G6P) if they were made heterotrophic by feeding sugars *in vitro* or when sugars accumulate in the cytosol *in vivo* (Quick et al., 1995; Lloyd and Zakhleniuk, 2004). It is now known that the hexose phosphate/phosphate (P_i_) translocators are part of a family of sugar phosphate and phosphoenolpyruvate translocators that include triose phosphate/P_i_ translocators, pentose phosphate/P_i_ translocators, and phosphoenolpyruvate/P_i_ translocators (Fischer et al., 1997; Kammerer et al., 1998; Hejazi et al., 2014). The hexose phosphate/P_i_ translocators (GPTs) work like the triose-phosphate/P_i_ translocators (TPTs). One molecule of hexose monophosphate is exchanged for one molecule of P_i_ or another sugar phosphate (Lee et al., 2017). The GPTs are promiscuous and will transport triose phosphates as well as, or better than, G6P, but GPTs will not transport G1P or F6P (Kammerer et al., 1998; Eicks et al., 2002).

The concentration of G6P in the chloplast and cytosol has been measured in the light five times in four species, spinach,potato, bean, and *Arabidopsis* and one species, bean, in the dark. In all cases the G6P concentration was found to be greater in the cytosol than the stroma both day and night (Gerhardt et al., 1987; Sharkey and Vassey, 1989; Schleucher et al., 1999; Weise et al., 2006; Szecowka et al., 2013). This makes it unlikely that expression of a glucose-6-phosphate/P_i_ translocator results in net G6P export from the chloroplast but rather G6P would be transported from the cytosol to the stroma. Two GPTs have been identified in *Arabidopsis*, *GPT1* At5g54800, and *GPT2* At1g61800 (Niewiadomski et al., 2005). *GPT1* has a low constitutive level of expression in almost all tissue and is necessary for embryo sac development and pollen maturation; *gpt1* mutants are embryo lethal (Niewiadomski et al., 2005). *GPT2* has low, almost undetectable, expression in all tissues but high expression amounts have been seen in leaves when plants are fed or accumulate sugars (Lloyd and Zakhleniuk, 2004; Dyson et al., 2014). Plants in which starch metabolism is compromised have higher expression of *GPT2* and chloroplast membranes are able to transport G6P (Kunz et al., 2010). *GPT2* transcript also increases in leaves in response to an increase in light and plays a role in the photosynthetic acclimation to high light (Athanasiou et al., 2010; Dyson et al., 2015).

Our goal was to better understand the regulation and metabolic role of GPT2 in day time metabolism. We measured transcript amounts using both qPCR and RNA-Seq of *GPT2* and other related genes in both the Columbia-0 and Wassilewskija ecotypes of *Arabidopsis* and a number of mutants under a variety of photosynthetic conditions. From this work, we found that *GPT2* is transcriptionally regulated and requires both triose phosphate export from the chloroplast and the Redox Responsive Transcription Factor 1 (RRTF1) for expression. We hypothesize that GPT2 plays an important role in facilitating starch synthesis in stochastic high light conditions and may be necessary to maintain the redox poise of the cytosol.

## Materials and Methods

### Plant Material and Growing Conditions

Wild-type *Arabidopsis thaliana* ecotype Columbia-0 (Col-0) and Wassilewskija (WS) and the mutants listed in Table 1 were used. All mutants used have been previously characterized with the exception of *tpt-3* SALK_028503. We found that the T-DNA insert was in the first exon based on the primer sites used to confirm the line was homozygous for the insert. Transcript of the *TPT* gene was checked by qPCR and was below our detection limit (results not shown).

**TABLE 1:** *Arabidopsis* mutants used in this study.

**Enzyme disrupted**	**Mutant**	**Gene locus**	**Ecotype**	**Mutant line and references**
Glucan water dikinase	*sex1-8*	At1g10760	Col-0	SALK_077211 (Ritte et al., 2006)
Plastid fructose 1,6-bisphosphatase	*hcef1*	At3g54050	Col-0	Livingston et al.,2010
Glucose-6-phosphate/P_i_ translocator 2	*gpt2-1*	At1g61800	Col-0	GABI_454H06 (Dyson et al., 2014)
Plastid phosphoglucomutase	*pgm1-1*	At5g51820	Col-0	TC75, *pgm1-1* (Egli et al., 2010)
Redox responsive transcription factor 1	*rrtf1-1*	At4g34410	Col-0	*Δrrtf1* SALK_150614 (Khandelwal et al., 2008)
Triose phosphate/P_i_ translocator	*tpt-3*	At5g46110	Col-0	SALK_028503 (This study)
Xylose-5-phosphate/P_i_ translocator	*xpt-2*	At5g17630	Col-3	SAIL_378_C01 (Hilgers et al., 2018)
Plastid phosphoglucomutase	*pgm1*	At5g51820	WS	ACG21 (Kiss et al., 1996)
Plastid starch phosphorylase	*phs1-1*	At3g29320	WS	Zeeman et al.,2004
Triose phosphate/P_i_ translocator	*tpt-1*	At5g46110	WS	Schneider et al.,2002

Plants were grown in a fluorescently lit growth chamber under a 12 h photoperiod with a photon flux density (PFD) of 120 μmol m^–2^ s^–1^. The daytime temperature was 23°C and the nighttime temperature was 20°C. Humidity was held at a minimum of 60% relative humidity. All plants used in this study were grown hydroponically to prevent drought stress and other stresses that might affect expression of genes being studied. The hydroponic setup used also minimized water vapor and CO_2_ loss from soil which can confound gas exchange measurements. Seeds were germinated on rockwool plugs which were placed in 1.5 ml microfuge tubes with the lower 1.5 cm tip cut off and watered with half-strength Hoagland’s solution (Hoagland and Arnon, 1938). Once roots started to grow through the bottom of the plug, the plants were transferred to a water culture hydroponic system in which plants were suspended over a vigorously aerated half-strength Hoagland’s solution contained in a dark tub. For all experiments, except RNA-Seq, data was collected from five biological replicates.

### Changing Light and CO_2_ Environments

Plants were placed so that their roots were suspended in a 50 ml disposable conical centrifuge tube containing half-strength Hoagland’s solution. The tube and plant were then placed in a LI-COR 6800 or 6400 portable gas exchange system (LI-COR Biosciences, Lincoln, NE, United States). Both systems used the small plant chamber lit with a LI-COR 6800-03 or LI-COR 6400-18A light source set to white light. Plants from each ecotype and mutant line were used in both systems to control for any system effect on gene expression. White light was maintained at a PFD of 120 μmol m^–2^ s^–1^ or 500 μmol m^–2^ s^–1^. Air temperature was held constant at 23°C and humidity was controlled at a dew point of 17 to 18.5°C. The CO_2_ concentration in the sample air, *C_*a*_*, was held at 20, 400, or 1000 ppm. A single leaf was taken from each plant after 0, 15, 30, 60, 120, and 240 min.

### Photosynthesis Measurements

For photosynthetic and fluorescence measurements a single leaf was placed in a LI-COR 6800-01A multiphase flash fluorometer gas exchange chamber. For these measurements, leaf temperature was held constant at 23°C and humidity was controlled at a dew point of 17 to 18.5°C. The lighting was 50% red and 50% blue actinic light which was provided by the LEDs in the fluorometer head. All fluorometer settings were left at the factory default. The relative electron transport rate was calculated as ΦPSII × I (light intensity) × α (leaf absorptivity assumed to be 0.85) × β (fraction of quanta absorbed by PSII, assumed to be 0.5). The numbers for α and β are the default parameters provided by LI-COR. Because these were not measured in our leaves the ETR is taken as a relative rather than absolute measurement.

### qPCR and Transcript Analysis

RNA was extracted using a Qiagen RNeasy Plant Mini Kit according to the manufacturer’s directions. Once RNA was isolated, cDNA was synthesized using 1 μg of total RNA. Invitrogen Super Script II reverse transcriptase (18064, Thermo Fisher Scientific, Waltham, MA, United States) was used according to manufacturer’s directions. An Eppendorf Mastercycler ep Realplex qPCR thermocycler with a 96 position silver block with SYBR green PCR master mix (4309155 Applied Biosystems, Carlsbad, CA United States) was used according to the manufacturer’s directions. The thermal profile was: 95°C for 10 min; 40 cycles of 95°C for 15 s, and 60°C for 1 min during which fluorescence was measured. This was followed by a melting curve of: 95°C for 15 s, 60°C for 15 s, a ramp from 60 to 95°C over a 20 min period during which time fluorescence was monitored, and 95°C for 15 s. Transcript amounts were normalized using the *ACTIN2* (*ACT2*) or *ISOPENTENYL DIPHOSPHATE ISOMERASE2* (*IDI2*) housekeeping genes. Absolute transcript amounts of these genes were checked during the 4-h time course in altered light or CO_2_ environments. This was done to ensure that the experimental treatment did not result in changes in housekeeping gene transcripts. Sequences for primers used are listed in Supplementary Table S1. Absolute copy number of transcripts was determined by using a slightly larger cDNA fragment of each target sequence as a standard. These cDNA standards were quantitated and dilutions were used to prepare standard curves.

### Statistics

Box plots and statistical differences in *GPT2* expression between WT and mutants were tested by one-way ANOVA followed by Tukey’s test using Microcal Origin 8.0 (Origin Lab Corporation, Northampton, MA, United States). Three levels of significance were tested and indicated by + α = 0.1; ^*^α = 0.05; ^∗∗^α = 0.01. Box plots are presented with the box encompassing the middle two quartiles, the mean shown as an open square inside the box, the median as a line inside the box, and the whiskers showing the standard error of the data.

### RNA-Seq Sampling and Sequencing

Three Col-0 and WS plants were used for RNA-Seq analysis and treated to a PFD of 500 μmol m^–2^ s^–1^ and sampled as above. All samples had an RNA integrity number of at least 7.0 as determined with a 2100 Bioanalyzer (Agilent Technologies, Santa Clara, CA, United States).

Single-end 50-bp mRNA sequencing was performed at the Michigan State University Research Technology Support Facility Genomics Core^1^. Libraries were prepared using the Illumina TruSeq Stranded mRNA Library Preparation Kit (Illumina, San Diego, CA, United States) following the manufacturer’s recommendations. Completed libraries were assessed for quality and quantified using a combination of *Qubit dsDNA* High Sensitivity Assay Kit (Qubit, Carlsbad, CA, United States), Caliper LabChipGX System and a DNA High Sensitivity Assay Kit (Caliper Life Sciences, Waltham, MA, United States), and Kapa Illumina Library Quantification qPCR assays (Illumina, San Diego, CA, United States). Each set of libraries were pooled in equimolar quantities and loaded on one lane of an Illumina HiSeq 2500 High Output flow cell (v4) (Illumina, San Diego, CA, United States). Sequencing was carried out in a 1 × 50 bp (SE50) format using HiSeq SBS reagents (v4) (Illumina, San Diego, CA, United States). Base calling was done by Illumina Real-Time Analysis (R-TA) v1.18.64 and output of RTA was demultiplexed and converted to FastQ format with Illumina Bcl2fastq v1.8.4. The average number of sequencing reads was 41.7 ± 11.2 million per sample.

### RNA-Seq Data Analysis

RNA-Seq data were subjected to gene DE analysis. Briefly, sequencing adapters and low quality bases were trimmed from sequencing reads using Trimmomatic version 0.32 (Bolger et al., 2014), and cleaned reads were examined using FASTQC software^2^ for quality evaluation. Reads were aligned to the reference genome, *A. thaliana* TAIR10 (Berardini et al., 2015), using the STAR (2.3.0e) alignment program (Dobin et al., 2013). The featureCounts function in the Rsubread (version 3) package for R was used to calculate read counts for each gene (Liao et al., 2013), and data were normalized as reads per kilobase of transcript per million mapped reads, (RPKM). We removed data for genes that had no reading higher that 7 RPKM and genes whose expression did not vary by more than two-fold (log_2_ < 1). Data represented in heat maps is after 15, 30, 60, 120, or 240 min at a PFD of 500 μmol m^–2^ s^–1^. For metabolic genes the data is the log_2_ of the ratio of the average RPKM value before and after light treatment. Data represented in heat maps of transcription factors is the log_2_ of the difference in the average RPKM value plus one before and after light treatment. Data for heat maps of transcription factors was calculated as a difference rather than a ratio because many transcription factors were not expressed or had expression amounts below our detection limit, RPKM = 0, before the light treatment. Heat maps were generated using the online Morpheus software^3^. The raw and processed data were deposited in the Gene Expression Omnibus (Edgar et al., 2002) and are accessible through GEO Series accession number GSEXXXXX^4^. (Raw RNA-Seq data is being submitted to NCBI, and accession numbers will be added during the revision process).

## Results

### *GPT2* Expression Is Related to Daytime Rather Than Nighttime Metabolism

**FIGURE 1:**
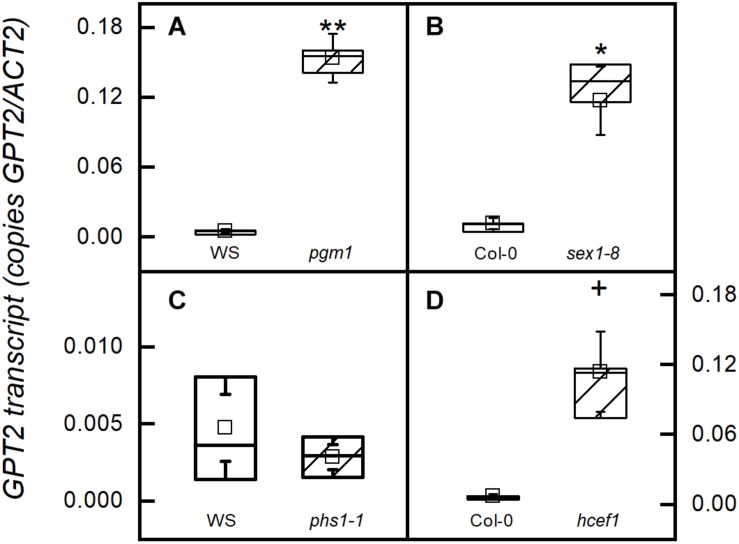
*GPT2* expression in starch related mutants. All samples were taken from plants in growth conditions, PFD 120 μmol m^–2^ s^–1^ and 400 ppm CO_2_. Transcripts were measured in **(A)** a starchless mutant (*pgm1-1*), **(B)** a starch excess mutant (*sex1-8*) that cannot degrade starch, **(C)** a starch phosphorylase knock out (*phs1-1*) that cannot carry out phosphorolytic starch degradation, and **(D)** a chloroplastic FBPase mutant (*hcef1*). ^*^α = 0.05; ^∗∗^α = 0.01; + α = 0.1, *n* = 5.

In mutants of *Arabidopsis* that are unable to synthesize starch due to a mutation in the gene encoding the plastid phosphoglucomutase (*pgm1-1*), *GPT2* transcripts amounts were more than two-fold higher than in the wild type WS (Figure 1A). A similar result is seen in a mutant lacking the glucan water dikinase 1 (*sex1-8*) (Figure 1B). In both the *pgm1-1* and *sex1-8* mutants starch synthesis does not occur in fully mature leaves. Although *sex1-8* mutant leaves have a lot more starch than wild-type, a threshold is reached at some point in the life cycle of the leaf and further accumulation does not occur (Caspar et al., 1991; Trethewey and ap Rees, 1994; Hejazi et al., 2014). However, when the ability to make G6P from starch degradation in the plastid is blocked by eliminating the plastidic starch phosphorylase (*phs1-1*), *GPT2* transcript amounts were unchanged (Figure 1C). *GPT2* transcript amounts were also elevated in a mutant lacking plastidic Calvin-Benson cycle enzyme fructose 1,6-bisphosphatase (*hcef1*) (Figure 1D). *GPT2* transcript increases when starch synthesis or early steps in the Calvin-Benson cycle are blocked. However, the lack of *GPT2* transcript when, G6P producing, phosphorolytic starch degradation is blocked, suggests a more significant role for GPT2 in daytime metabolism.

### *GPT2* Is Expressed When Photosynthesis Is Increased by Light

**FIGURE 2:**
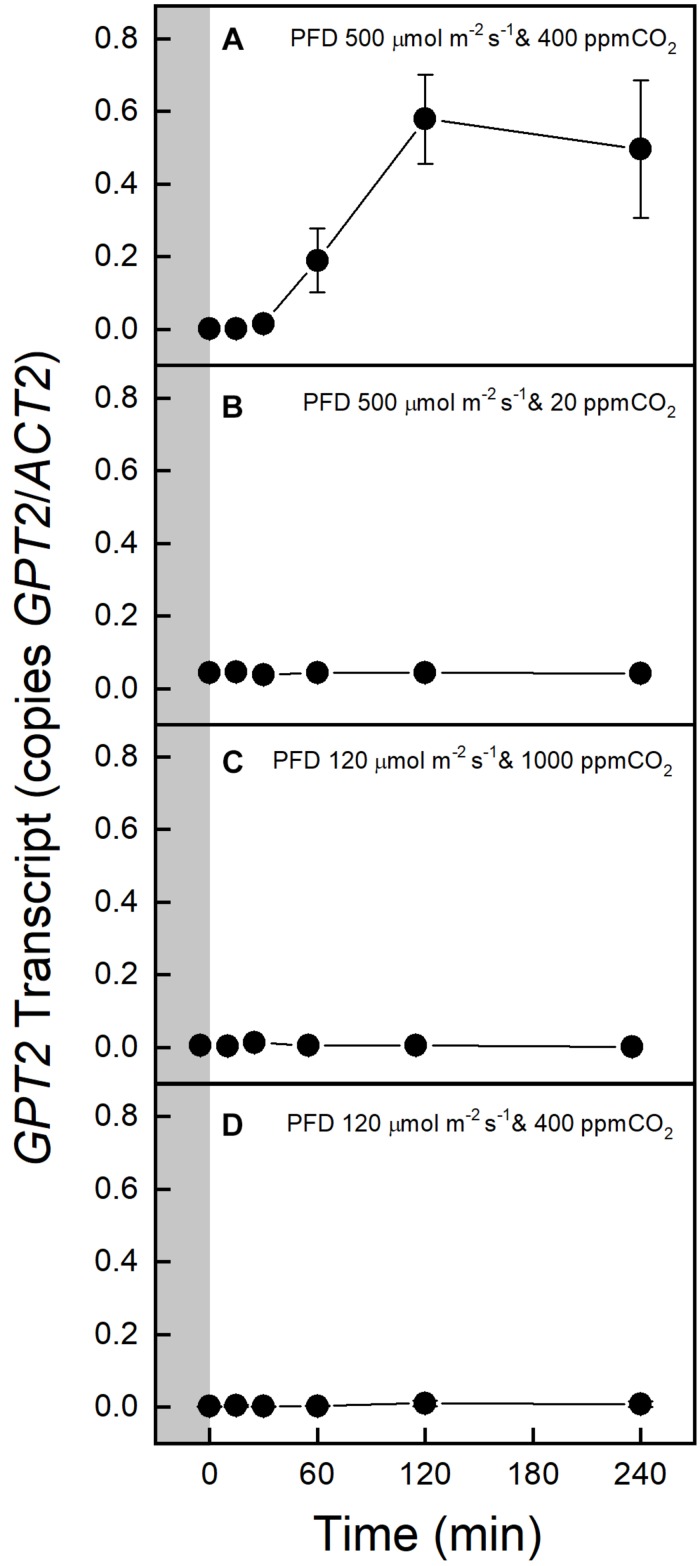
Expression of *GPT2* in Col-0 before and after transfer to an altered light or CO_2_ environment. The gray block indicates sampling in growth conditions, PFD 120 μmol m^–2^ s^–1^ and 400 ppm CO_2_. Panels **A–D** show responses to various light and CO_2_ treatments as indicated on the figure. *n* = 5.

A switch from a PFD of 120 to 500 μmol m^–2^ s^–1^ increased *GPT2* expression in Col-0, but 500 μmol m^–2^ s^–1^ of light with photosynthesis restricted by 20 ppm CO_2_ did not result in increased expression (Figures 2A,B). 1000 ppm CO_2_ alone did not result in an increase in *GPT2* transcript (Figure 2C). Plants were also placed in the gas exchange system at growth conditions of 120 μmol m^–2^ s^–1^ of light and 400 ppm CO_2_ to make sure that moving the plants into the gas exchange system for 4 h did not result in an increase in transcript (Figure 2D). The same patterns were observed in the WS ecotype (Supplementary Figure S1).

**FIGURE 3:**
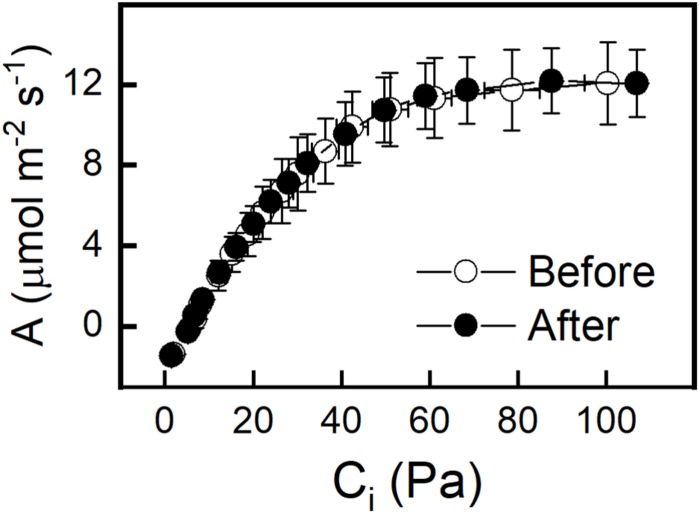
A – C_i_ curves of Col-0 taken before and after 4 h incubation at a PFD of 500 μmol m^–2^ s^–1^ and C_a_ of 400 ppm. A – C_i_ curves were done at a PFD of 500 μmol m^–2^ s^–1^. *n* = 5.

The effect of an increased PFD of 500 μmol m^–2^ s^–1^ on photosynthesis was determined by taking *A-C_*i*_* curves before and after the 4-h light treatment. No change was observed in the Col-0 or WS ecotype, indicating that there were no significant non-photochemical processes or photo damage (Figure 3 and Supplementary Figure S2). To better understand why increased light but not increased CO_2_ resulted in an increase in *GPT2* transcript both photosynthesis and relative electron transport rates (ETR) were measured at a variety of different PFDs and CO_2_ concentrations. The relative electron transport rate divided by four was taken as an approximate turnover rate of the Calvin-Benson cycle. This is because four electrons are used per two NADPH needed for RuBP regeneration. Increasing the PFD from 120 to 500 μmol m^–2^ s^–1^ increased the photosynthetic rate by 86% and increased the Calvin-Benson cycle turnover by 122% (Supplementary Figures S3A,B). Increasing CO_2_ alone caused a 40% increase in assimilation but only a 1.4% increase in Calvin-Benson cycle turnover (Supplementary Figures 3C,D). The increase in light resulted in a much larger increase in photosynthesis and Calvin-Benson cycle turnover than did an increase in CO_2_.

### *GPT2* and *XPT* Expression Are Not Affected by Each Other

The XPT may transport pentose phosphates into the chloroplast from the cytosolic oxidative pentose phosphate pathway (cytosolic G6P shunt). *GPT2* transcript was measured in the *xpt-2* mutant. No significant difference was observed between the *xpt-2* mutant and wild type in response to an increased PFD of 500 μmol m^–2^ s^–1^ (Supplementary Figure S4A). There was also no difference in *GPT2* expression when the plastidic starch phosphorylase (*phs1-1*) was missing (Supplementary Figure S4B). Conversely *XPT* transcript was measured in response to increased light in the *gpt2-1* mutant. There was no transcriptional evidence for XPT compensating for GPT2. Transcript amount of *XPT* was low and unchanged and was also unchanged in the *phs1-1* mutant background (Supplementary Figures S4C,D).

### Starch Synthesis Is Upregulated While Other Pathways Are Downregulated

An RNA-Seq analysis was performed to examine changes in the entire transcriptome when plants were taken from low to high light. RNA was taken from leaves after they were exposed to high light at ambient CO_2_ conditions for 15, 30, 60, 120, and 240 min and the ratio in transcript amount from the zero time point (low light, ambient CO_2_) was determined. Genes coding for enzymes in the Calvin-Benson cycle, the chloroplast G6P shunt, starch synthesis, sucrose synthesis, and cytosolic glycolysis were identified. A complete list of the gene loci for biochemical pathways of interest was collated (Supplementary Appendix S1). From these lists, only transcripts with an RPKM value greater than 7 and an absolute fold change greater than 2 (log_2_ value > | 1|) at any one time point were examined. The *Arabidopsis* gene loci from Supplementary Appendix S1 that were identified as being differentially expressed are listed in Supplementary Table S2.

**FIGURE 4:**
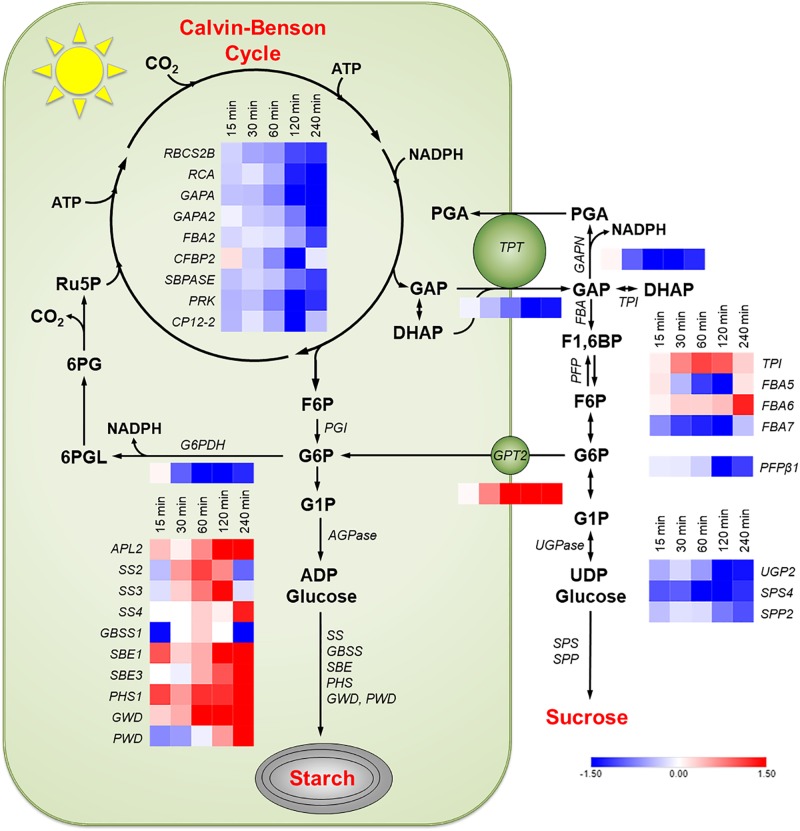
Changes in transcription in Col-0 in response to an increase in PFD from 120 to 500 μmol m^–2^ s^–1^. Transcripts of the following pathways or genes are shown: Calvin-Benson cycle, starch synthesis, sucrose synthesis, the G6P shunt, the non-phosphorylating GAPDH (*GAPN*) in the cytosol, the glucose-6-phosphate transporter (*GPT2*), and the triose phosphate transporter (*TPT*). Red boxes represent an increase in transcription and blue a decrease. Expression values shown had a log_2_ RPKM fold change greater than one and are on an absolute scale of –1.5 to 1.5. *n* = 3.

Transcripts for every enzyme in the Calvin-Benson cycle and of genes coding for electron transport proteins, identified by Dyson et al. (2015), decreased during the 240 min high light treatment. The transcripts meeting the cutoff stated above for enzymes in the Calvin-Benson cycle are shown in Figure 4. There was a large increase in transcript amounts for most enzymes involved in starch synthesis (Figure 4). There was a steep decline in transcripts for the most highly expressed sucrose synthesizing enzymes sucrose-phosphate synthase 4 (SPS4) and sucrose-phosphate phosphatase 2 (SPP2) (Figure 4). *GPT2* transcript was much higher in the RNA-Seq experiment as had been seen with qPCR (Figure 2A). The transcript amount for the *TPT* decreased by 15 min and was down 65% after 120 min. The same pattern of changes was observed in the WS ecotype (Supplementary Figure S5).

**FIGURE 5:**
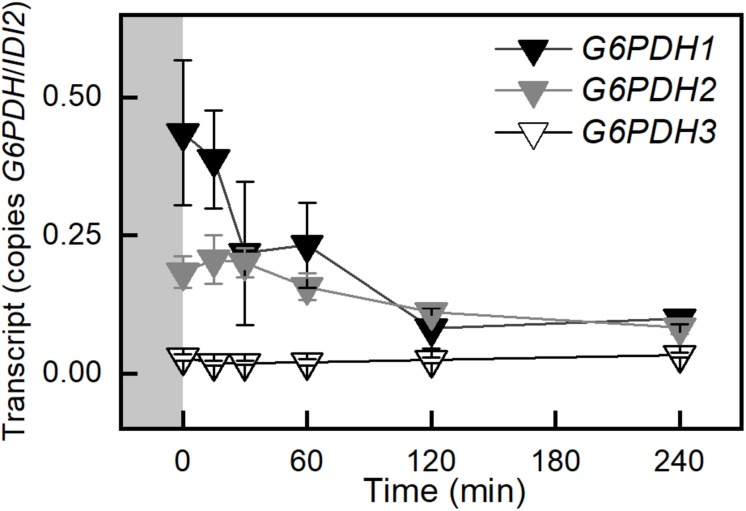
Changes in transcription of the plastid *G6PDH*s in Col-0 in response to an increase in PFD from 120 to 500 μmol m^–2^ s^–1^ C_a_ 400 ppm. Gray block indicates sampling in growth conditions, PFD 120 μmol m^–2^ s^–1^ and 400 ppm CO_2_. *n* = 5.

Transcript amounts for proteins involved in the cytosolic glycolysis pathway were examined. Most genes in this pathway were not differentially expressed. Two uncharacterized pyruvate kinases, At5g63680, and At3g52990 had increased transcript amounts (data not shown). *Arabidopsis* has three glyceraldehyde phosphate dehydrogenases in the cytosol. The only one that was differentially expressed in response to high light was the non-phosphorylating glyceraldehyde-3-phosphate dehydrogenase (GAPN). *GAPN* transcript was down 56% after 120 min (Figure 4). This pattern was also observed in the WS ecotype (Supplementary Figure S5). G6P shunt (Sharkey and Weise, 2016) transcripts were also examined in Col-0 and WS. Transcript for *glucose-6-phosphate dehydrogenase 1* (*G6PDH*) decreased by 87% (Figure 3 and Supplementary Figure S6). This decrease was validated by qPCR. *G6PDH2* also decreased and *G6PDH3* had a very low and unchanging expression amount (Figure 5 and Supplementary Figure S6).

Transcript amounts for proteins involved in protecting photosynthetic electron transport and PSII repair were examined to determine if light stress was a factor in these experiments. Transcripts of eight genes were differentially expressed according to the cutoff criteria stated above. From this group of eight, seven had decreasing transcript amounts and only one PSII assembly factor *Slr1768* At5g51570 (Nickelsen and Rengstl, 2013), had an approximate 2.7 fold increase after 4 h (data not shown). Because there was no decrease in photosynthesis after 4 h at a PFD of 500 μmol m^–2^ s^–1^ and transcript of only one gene for PSII repair increased, it was concluded that light stress was not a significant factor.

### Transcripts for 30% of Transcription Factors Were Differentially Expressed

Transcript amounts were examined from the RNA-Seq data of all genes identified as transcription factors or possible transcription factors by the *Arabidopsis* Gene Regulatory Information Server (AGRIS^5^). From this initial pool of 1823 genes 183 were discarded because their sequences were not detected in this experiment. Four hundred and 84 genes, 30%, were selected that had a change in the RPKM value (ΔRPKM) between zero and either 15, 30, 60, 120, or 240 min time points in either Col-0 or WS that was greater than or equal to seven. This cutoff eliminated as many non-responding transcription factors as possible while still containing 15 of the 19 transcription factors that were identified by Vogel et al. (2014) as being quickly upregulated in response to high light and involved in retrograde, chloroplast to nucleus, signaling. From this pool 227 and 209 transcription factors, 13%, in Col-0 and WS, respectively, were selected that showed increased expression in response to light. Increased expression was defined as transcripts with a RPKM value that increased from the initial time zero value by two or more at any of the sampling time points and did not decrease at any time point by more than the initial RPKM value minus two. These pools contained 11 and 10 of the Vogel et al. (2014) transcription factors in Col-0 and WS, respectively.

**FIGURE 6:**
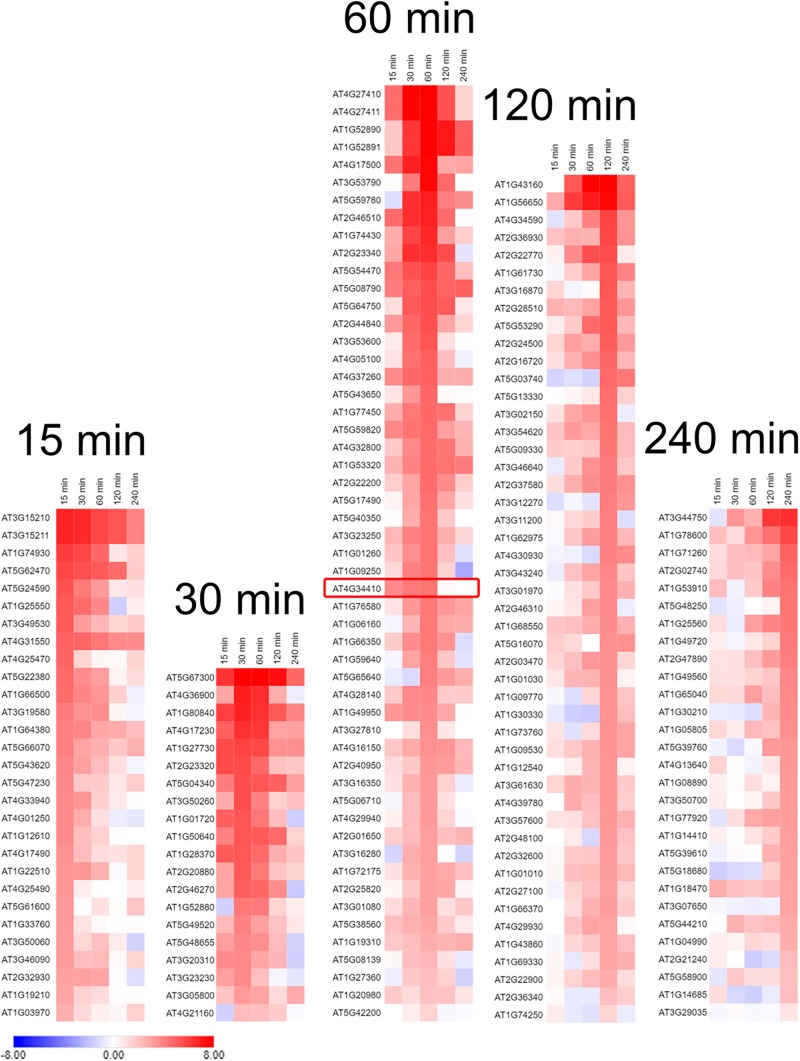
Transcription factors in Col-0 whose expression was increased in response to an increase in PFD from 120 to 500 μmol m^–2^ s^–1^. Transcription factors are grouped according to the time they had the highest transcript abundance after transfer to high light. The red box is around the *RRTF1* transcription factor. Expression values used for heat map generation are the log_2_ ΔRPKM + 1 and are on an absolute scale of –8 to 8. *n* = 3.

Waves of increased expression of transcription factors were observed between the 15 and 240 min sampling time points (Figure 6 and Supplementary Figure S5). Transcription factors that had a maximal transcript abundance at the 30 or 60 min sampling time points were of particular interest because they preceded the maximal abundance of *GPT2* at 120 min (Figures 2, 4). From this list of 19 transcription factors identified by Vogel et al. (2014) the *RRTF1* had the highest transcript abundance at the 30 or 60 min sampling time (Figure 6 and Supplementary Figure S7).

### *GPT2* Expression Is Repressed in the *tpt-3* and *rrtf1-1* Mutants

**FIGURE 7:**
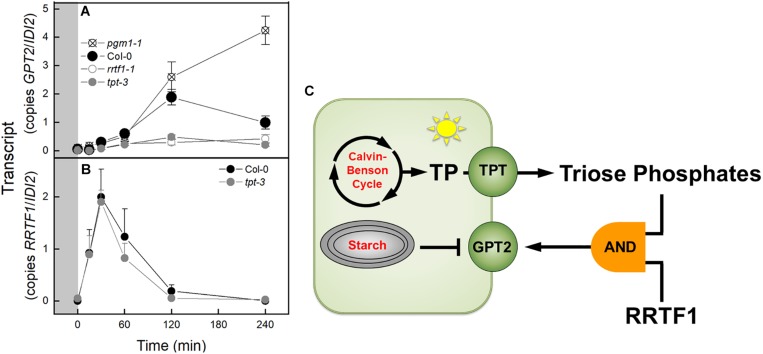
Expression of *GPT2* and *RRTF1* in response to an increase in PFD from 120 to 500 μmol m^–2^ s^–1^ C_a_ 400 ppm in Col-0 and mutants and model of transcriptional regulation of *GPT2*. **(A)** qPCR data of expression of *GPT2* in Col-0 and *pgm1-1*, *rrtf1-1*, and *tpt-3* mutants. *n* = 5 **(B)** qPCR data of expression of *RRTF1* in Col-0 and *tpt-3* mutant. *n* = 5 **(C)** Model of transcriptional regulation of *GPT2* expression. Triose phosphates in the cytosol and *RRTF1* are both required for expression of *GPT2* (shown as an “AND” logic gate) while starch synthesis represses *GPT2* expression.

Based on the results of transcript analysis by qPCR or RNA-Seq the expression of *GPT2* or *RRTF1* was examined in various mutant backgrounds. *GPT2* transcript was elevated 34 fold in the *pgm1-1* mutant at time 0 in a PFD of 120 μmol m^–2^ s^–1^ (Figure 7A). This was similar in magnitude to the increased *GPT2* expression in the *pgm1-1* mutant observed in Figure 1. This 34 fold increase in *GPT2* was dwarfed by the 1,321 fold increase observed after 4 h at a PFD of 500 μmol m^–2^ s^–1^ and thus cannot be seen in the figure (Figure 7A). In Col-0 or WS after 240 min the expression of *GPT2* was attenuated. This attenuation did not occur in *pgm1-1* mutant plants with transcript amounts of 120% higher than WT (Figure 7A). In plants missing the TPT, the increase in expression of *GPT2* in response to high light was reduced by 74% (Figure 7A). This was also observed in a *tpt-1* mutant line in the WS background (Supplementary Figure S8A). *GPT2* expression was reduced in *rrtf1-1* mutant plants (Figure 7A). To determine if triose phosphates in the cytosol controlled the expression of *RRTF1*, *RRFT1* expression was measured in *tpt-3* mutant plants. *RRTF1* transcript increased in response to high light in the *tpt-3* mutant to the same degree as WT in with the Col-0 and WS backgrounds (Figure 7B and Supplementary Figure S8B). The pattern of *RRTF1* expression was the same as was observed in the RNA-Seq data.

## Discussion

### Expression of GPT2 Requires Triose Phosphate Export and RRTF1

Expression of both *TPT* and *RRTF1* are necessary for the increased expression of *GPT2*. If either one of these is missing transcript of *GPT2* does not increase in response to an increase in light. The lack of *GPT2* transcript response to an increase in light in a *tpt* mutant has been seen before (Kunz et al., 2010; Heinrichs et al., 2012). This is presumably due to low triose phosphate concentration in the cytosol. The triose phosphates themselves could act as a signal (Häusler et al., 2014). Another possibility is that the sucrose generated from the triose phosphates is acting as a signal. In similar light jump experiments to ours, Schmitz et al. (2014) measured sucrose amounts in Col-0 and the *adg1-1* starchless mutant of *Arabidopsis* for 8.3 h after being transferred from a PFD of 30 to 300 μmol m^–2^ s^–1^. The pattern of sucrose accumulation in the leaves mirrors the pattern of *GPT2* transcript seen in WT and the *pgm1-1* mutant. Sucrose would present a more stable signal than rapidly fluctuating triose phosphate amounts and signaling by sucrose via invertases and hexokinase is well documented (Li and Sheen, 2016).

A third possibility is that a change in export of triose phosphates could cause change in the redox status of the cytosol. Glyceraldehyde 3-phosphate (GAP) could be converted to phosphoglycerate (PGA) by the non-phosphorylating glyceraldehyde-3-phosphate dehydrogenase (GAPN) resulting in the production of NADPH in the cytosol (Habenicht, 1997). The PGA could then be reimported to the chloroplast via the TPT or further metabolized and imported as PEP, or pyruvate. This could result in a change in the redox poise of the cytosol that is concomitant with a change in triose phosphate export. This change in redox could be the signal that activates *RRTF1* (Vogel et al., 2014). However, based on our results, *RRTF1* is induced independently of triose phosphate concentration in the cytosol. The increase in *RRTF1* transcript in the *tpt-3* mutant (Figure 7B) was not seen by Vogel et al. (2014). After carefully scrutinizing the methods of the Vogel et al. (2014) study the reason for this difference is unclear.

There are likely other transcription factors that influence GPT2 expression. Our RNA-Seq results revealed over 70 transcription factors with a similar transcript profile to *RRTF1* (Figure 6). Further the regulation of *GPT2* transcript by RRTF1 may be indirect. However, RRTF1 was of particular interest because of its identification by Vogel et al. (2014) as early acting – minutes as opposed to days – in response to increased light and to be involved in retrograde, chloroplast to nucleus, signaling. *RRTF1* had the highest expression of the 19 transcription factors identified by Vogel in the 30–60 min window preceding the rise in *GPT2* expression. To determine if the interaction between *GPT2* and RRTF1 is direct, experiments could be done in which the promoter of GPT2 is tested for interaction with RRTF1 protein using a yeast one-hybrid system or a plant cell GUS-dependent assay (Berger et al., 2007). Based on our results with both *tpt-3* and *rrtf1-1* mutants we conclude that at least both TPT and RRTF1 are required for expression of *GPT2*, the two acting as inputs to a logic “AND” gate (Figure 7C).

When starch metabolism is blocked, *GPT2* transcript continues to increase in response to increased light but in WT, *GPT2* transcript amount declines after 4 h (Figure 7A). This attenuation could be caused by a reduction of triose phosphate entry into the cytosol. It could also be caused by an increase in triose phosphate conversion to hexose phosphate and re-import into the chloroplast and starch synthesis. This possibility is consistent with the increase in TPI and FBP aldolase (FBA6) expression (Figure 4). Transcript amounts for enzymes in starch synthesis increased after 15 min of higher light and an increase in protein and activity would be possible after 2- 4 h (Figure 4 and Supplementary Figure S5), when a drop in *GPT2* transcript is observed (Figure 2A and Supplementary Figure S1A).

GPT2 could play an important role in controlling the source-sink balance of triose phosphates in the cytosol. When there is a rapid increase in the photosynthetic rate, caused by light, transcripts of genes related to sugar phosphate anabolism might be expected to increase. However, there is a large decline in transcripts of genes in the Calvin-Benson cycle and of the triose phosphate transporter. This leads to the hypothesis that unregulated triose phosphate production and export are a problem for the plant. *GPT2* expression could lead to more GPT2 protein in the chloroplast envelope. This may result in a larger plastid sink for hexose phosphate and in turn a greater proportion of triose phosphates being converted to hexose phosphates and used in starch synthesis. This would decrease the amount of GAP that could be acted upon by GAPN (Figure 4, Pathway) and prevent an over reduction of the cytosol.

Starch and sucrose are the two largest end products of photosynthesis (McClain and Sharkey, 2019). When photosynthesis increases, an increase in transcript amounts for enzymes in both starch and sucrose synthesis could be expected. However, a reduction in expression of three critical genes involved in sucrose synthesis was observed (Figure 4 and Supplementary Figure S5). In the process of acclimation, days as opposed to hours, an increase in enzymes related to sucrose synthesis has been observed in response to increased light (Miller et al., 2017). In the short term, there is generally a stronger increase of transcripts for processes leading from triose phosphates to hexose phosphate with triose phosphate isomerase standing out, having over a twofold increase in transcript amounts 60 min after being transferred to high light. Changes in expression related to G6P metabolism and transport may serve two functions. It may increase starch synthesis by bypassing the plastidic PGI, or it may provide an additional sink for triose phosphates so that GAP is not converted to PGA by GAPN, disturbing the redox poise of the cytosol.

### Decreasing Transcript for G6PDH1 May Limit Carbon Entry Into the G6P Shunt

The import of G6P via GPT2 could be problematic because it could also be acted on by glucose-6-phosphate dehydrogenase (G6PDH) in the chloroplast (Preiser et al., 2019). G6PDH is generally considered to be deactivated in the light when the chloroplast stroma is reduced. During the day, when the stroma is reduced, the *K_*m*_* of G6PDH increases, but some residual activity remains (Scheibe et al., 1989; Hauschild and von Schaewen, 2003; Preiser et al., 2019). It has been shown that this inhibition of activity can be reversed in bacteria by low mM concentrations of G6P (Cossar et al., 1984). This would result in the G6P entering the G6P shunt leading to a futile cycle consuming ATP and releasing CO_2_ (Sharkey and Weise, 2016; Preiser et al., 2019). Under certain circumstances this shunt may be adaptive allowing the generation of pentose phosphates to restart the Calvin-Benson cycle when triose phosphates are depleted. It could also mitigate a high ATP/NADPH ratio under conditions of high cyclic electron transport (Sharkey and Weise, 2016). *Arabidopsis* has three G6PDH enzymes in the chloroplast, G6PDH1, *2*, and *3* (Wakao and Benning, 2005). Transcript for G6PDH1 (Figure 5 and Supplementary Figure S6), and to a lesser extent G6PDH2, rapidly decrease after the light is increased. It has been proposed that these two contribute the majority of activity for the chloroplast G6PDHs (Wakao and Benning, 2005). One interpretation of the data is that expression of *GPT2* in response to increased light leads to an increase in G6P concentration in the stroma and the reduction in expression of *G6PDH1* and *2* lead to reduced G6PDH activity, limiting the flow of carbon into the G6P shunt.

### GPT2 Expression May Reflect Daytime Metabolic Needs Rather Than Nighttime

Mutants of *Arabidopsis* that are unable to make starch due to a mutation in the plastidic *PGM* have elevated transcript for *GPT2* (Figure 1). The effect of mutations in genes required for starch synthesis on *GPT2* expression has been seen before in mutants in *PGI1*, *PGM1*, and *AGPase* (Bläsing et al., 2005; Kunz et al., 2010). A mutant with a T-DNA insert in the gene for the glucan water dikinase enzyme, the *sex1-81* mutant, can make starch but cannot use it (Ritte et al., 2002). An increase in *GPT2* transcript is also seen in this mutant. The starch phosphorylase, *phs1-1*, mutant does not have elevated *GPT2* transcript (Figure 1). Because of this the increase in *GPT2* transcript in mutants unable to make or mobilize starch is probably not caused by a depletion of hexose phosphates at night. Additionally, it has been shown that *GPT2* transcript in starchless mutants is repressed in the dark (Kunz et al., 2010).

The increase in *GPT2* transcript in the starchless or starch excess mutants could be the result of increased triose phosphates in the cytosol that are not being partitioned into starch. It has been suggested that in starchless mutants, GPT2 may serve to export G6P from the chloroplast (Kunz et al., 2010). In a wild type plant, export of G6P would be unlikely due to the large concentration gradient of G6P measured between the cytosol and chloroplast (Gerhardt et al., 1987; Weise et al., 2006; Szecowka et al., 2013). The concentration difference of G6P between the chloroplast and cytosol in starchless mutant or starch excess mutants is unknown. GPT2 can transport triose phosphates as well as or better than hexose phosphates (Kammerer et al., 1998; Eicks et al., 2002) leaving open the possibility that GPT2 simply serves as an additional export mechanism for triose phosphates in the starchless background.

### *GPT2* Expression Allows a Bypass of Calvin-Benson Cycle FBPase

When the Calvin-Benson cycle enzyme fructose-1,6-bisphosphatase (FBPase) is missing, *GPT2* transcript amounts are increased (Figure 1). The cytosolic FBPase could be used, bypassing the stromal FBPase when GPT2 is present. This comes at a cost of growth and increased cyclic electron transport (Livingston et al., 2010). The chloroplast PGI favors the reaction from F6P to G6P (Preiser et al., 2018). Therefore, *GPT2* expression could result in the chloroplastic G6P concentration being higher than normal. This G6P could be acted on by G6PDH and enter the G6P shunt, detailed above. Any increase in the G6P shunt would result in the need for extra ATP which could be provided by cyclic electron transport. A similar scenario has been seen in *Arabidopsis* plants in which the plastidic triose phosphate isomerase is inhibited by high concentrations of 2-phosphoglycolate. These plants also have elevated expression of *GPT2* and have higher cyclic electron transport (Li et al., 2019). Plants lacking the stromal FBP aldolase have higher cyclic electron flow consistent with this hypothesis (Gotoh et al., 2010) although it is not known if they have increased expression of *GPT2.* In another study using a chloroplast FBPase mutant, elevated *GPT2* transcript was seen but only in the roots (Soto-Suárez et al., 2016). The reasons for the spatial difference in *GPT2* expression observed between our study and that one are unclear. Another possible explanation for the survival of the *hcef1* mutant is a novel redox insensitive plastid FBPase that was found in strawberry but appears to have an ortholog in *Arabidopsis* (Serrato et al., 2009). This use of an alternative FBPase does not explain the increased *GPT2* transcript and high cyclic ETR seen in the *hcef1* mutant.

Another possible pathway for carbon exit and reentry into the chloroplast is the oxidative pentose phosphate pathway in the cytosol. It has been shown that the cytosolic G6PDH in potato is sensitive to the sugar status of the cell and is transcriptionally regulated (Hauschild and von Schaewen, 2003). Using this pathway, carbon could reenter the chloroplast though the xylulose-5-phosphate transporter (XPT). If this pathway is used there is no evidence for transcriptional regulation of it. The transcript amount for XPT was unchanged in the wild type, *gpt2-1* or *phs1-1* mutants (Supplementary Figure S4). Further, no change in transcript was seen in the RNA-Seq data for XPT or any enzyme, including the three G6PDHs in the cytosolic pentose phosphate pathway (data not shown).

### Only an Increase in Calvin-Benson Cycle Activity Caused *GPT2* Transcript to Increase

Increasing CO_2_ has been shown to increase the expression of a homologous gene to *GPT2* in soybean in a Free Atmosphere CO_2_ Enrichment (FACE) experiment (Leakey et al., 2009). In our experiment, CO_2_ was increased under a PFD of 120 μmol m^–2^ s^–1^ rather than the higher PFD of the FACE site field conditions. No change in *GPT2* expression was observed (Figure 2C and Supplementary Figure S1C). Under these conditions the assimilation rate and electron transport rate increased only modestly (Supplementary Figure S3). This most likely did not result in a large change in cytosolic triose phosphate or sucrose concentration or redox status that appears likely to be the drivers of *GPT2* expression.

## Conclusion

Glucose-6-phosphate/phosphate translocator 2 is at the confluence of metabolic adjustment to sudden increases in carbon assimilation. While normally not expressed, *GPT2* transcript rapidly increases when the rate of photosynthesis increases. The time course of RNA-Seq results after an increase in photosynthesis provides valuable insights into the metabolic tradeoffs plants make. When photosynthesis increases, GPT2 may act as a safety-valve, shunting sugar phosphates away from the NADPH-producing non-phosphorylating GAPDH. At the same time GPT2 can also shunt extra carbon toward starch synthesis, bypassing the regulatory stromal PGI. Connecting the cytosolic and stromal G6P pools comes at a cost. The increase in G6P in the chloroplast may activate the stromal G6PDH leading to loss of ATP and loss of carbon, when 6-phosphogluconate dehydrogenase in the G6P shunt releases CO_2_. However, the reduction in expression of genes related to sucrose synthesis is puzzling.

## Data Availability

The datasets generated for this study can be found in Gene Expression Omnibus. The raw and processed data were deposited in the Gene Expression Omnibus (Edgar et al., 2002) and are accessible through the GEO Series accession number GSE132626 (http://www.ncbi.nlm.nih.gov/geo/query/acc.cgi?acc=GSE132626) (Raw RNA-Seq data is being submitted to the NCBI, and accession numbers will be added during the revision process).

## Author Contributions

SW and TS designed the experiments. SW carried out the bulk of the research. KC and TL carried out the RNA-Seq and the initial analysis of RNA-Seq data. AP, HK, and CP-P worked out some of the qPCRs and analyzed the results. SW wrote the manuscript. TS edited the manuscript.

## Conflict of Interest Statement

The authors declare that the research was conducted in the absence of any commercial or financial relationships that could be construed as a potential conflict of interest.

## Footnotes

1 https://rtsf.natsci.msu.edu/genomics/

2 https://www.bioinformatics.babraham.ac.uk/projects/fastqc/

3 https://software.broadinstitute.org/morpheus

4 http://www.ncbi.nlm.nih.gov/geo/query/acc.cgi?acc=GSEXXXXX

5 https://agris-knowledgebase.org/
