# Supplementary material for: Transcriptional Regulation of the Glucose-6-Phosphate/Phosphate Translocator 2 Is Related to Carbon Exchange Across the Chloroplast Envelope
Source: Front Plant Sci. 2019 Jun 27;10:827. doi: 10.3389/fpls.2019.00827 (PMC6609314; doi:10.3389/fpls.2019.00827)
Supplement: Supplementary file 1 [file Presentation_1.pdf]

Transcriptional regulation of the glucose-6-phosphate/Phosphate translocator 2 is related to carbon exchange across the chloroplast envelope

Supplementary Tables and Figures

Sean E. Weise[1,2], Tiffany Liu[3], Kevin L. Childs[3], Alyssa L. Preiser[1], Hailey M. Katulski[1], Christopher Perrin-Porzondek[1], Thomas D. Sharkey[1,2,4 *]

[1]MSU-DOE-Plant Research Laboratory, Michigan State University, East Lansing, MI USA
[2]Department of Biochemistry and Molecular Biology, Michigan State University, East Lansing, MI USA
[3]Department of Plant Biology, Michigan State University, East Lansing, MI USA
[4]Plant Resilience Institute, Michigan State University, East Lansing, MI USA

Supplementary Table S1. Primer sequences used in this study

Gene	Gene Locus	Standard Generating Primers	qPCR primers
ACT2	At3g18780	GGTGATGAAGCACAATCCAA<br>CAGTAAGGTCACGTCCAGCA	CAAAGGCCAACAGAGAGAAGA<br>ATCACCAGAATCCAGCACAA
IDI2	At3g02780	CCGAATTTTCGTGCTTTCTC<br>TGCAGCATTCCTCACACCTA	GTATGAGTTGCTTCTCCAGCAAAG<br>GAGGATGGCTGCAACAAGTGT
GPT2	At1g61800	CGTGGAAGGTCCTCAAATGT<br>TCACTGCTTCGCCTGTGAGT	AGACCAGATTTCGCCGTTAACT<br>ACTGCTTCGCCTGTGAGTAGAG
XPT	At5g17630	AGGATCACATTGGGTTCCAG<br>CTTTGCAGTGGCCTGAGAAT	CATTGCATCTGTTGGGACAC<br>TGGACTGATCTCGTCGAGTG
G6PDH1	At5g35790	GAGAATGGCTGGACAAGGGT<br>GCCGGTGTGAATAGATCCCA	ATTTCGGAACAGAAGGGCGT<br>CAACCACATCTTCAAGCCGC
G6PDH2	At5g13110	TGGAAGAATCTCAAACCGCCT<br>AGCCTCTCATATGCATCTGGT	ATTTCGGCACTGAAGGACGT<br>GCGTTTCCATGGCAAAGAGG
G6PDH3	At1g24280	GAGTCTGATGGCGGTGAACA<br>CTGCAAATGTCGGGGTCAGA	TGCATTGGACGAGAAGCTCA<br>CACATTTCACCGCGTCAACA
RRTF1	At4g34410	CCCAACCCGGTATCAAAAGGA<br>CTTGGCCCACGGAATCCAAT	GCCTCGGTTGGATTCAGACA<br>TGATTTTCCCGCCACCTTCC

Supplementary Table S2. Specific differentially expressed *Arabidopsis* genes and loci used in heat maps in figure 4 and supplementary figure S5.

Gene	Locus	Gene	Locus
**Calvin-Benson Cycle**		**TP to G6P**	
RBCS1A	At1g67090	*TPT*	At5g46110
RBCS1B	At5g38430	*TPI*	At3g55440
RBCS2B	At5g38420	*FBA5*	At4g26530
RBCS3B	At5g38410	*FBA6*	At2g36460
RCA	At2g39730	*FBA7*	At4g26520
GAPA	At3g26650	*GPT2*	At1g61800
GAPA2	At1g12900	**G6P to TP**	
FBA2	At4g38970	*PFPβ1*	At4g04040
CFBP2	At5g64380	**G6P to Sucrose**	
SBPASE	At3g55800	*UGP2*	At5g17310
PRK	At1g32060	*SPS3*	At1g04920
CP12-2	At3g62410	*SPS4*	At4g10120
**Starch Synthesis**		*SPP2*	At2g35840
APL2	At1g27680	*G6PDH1*	At5g35790
APL3	At4g39210	*G6PDH2*	At5g13110
APL4	At2g21590	*G6PDH3*	At1g24280
SS2	At3g01180	*GAPN*	At2g24270
SS3	At1g11720		
SS4	At4g18240		
GBSS1	At1g32900		
SBE1	At3g20440		
SBE3	At2g36390		
PHS1	At3g29320		
GWD	At1g10760		
PWD	At5g26570		

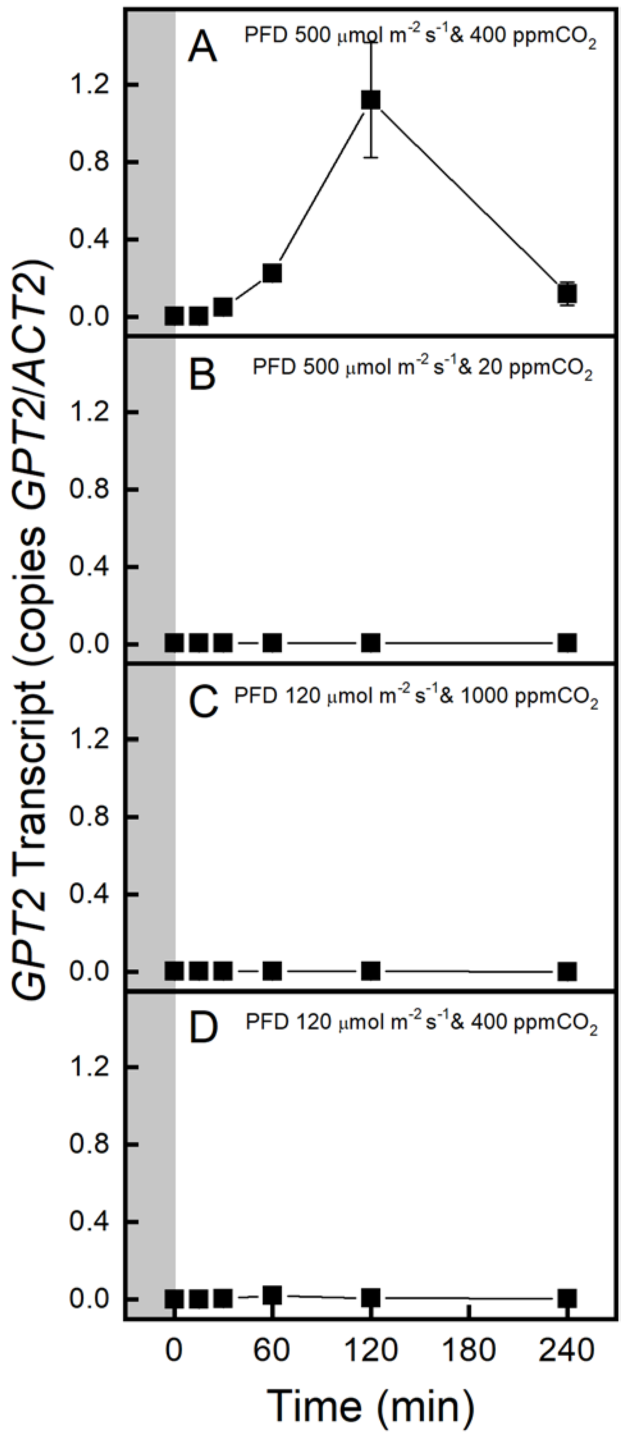

Supplementary Figure S1. Expression of *GPT2* in WS before and after transfer to an altered light or CO_2 environment. The grey block indicates sampling in growth conditions, PFD 120 μmol m^{-2} s^{-1} and 400 ppm CO_2. n = 5

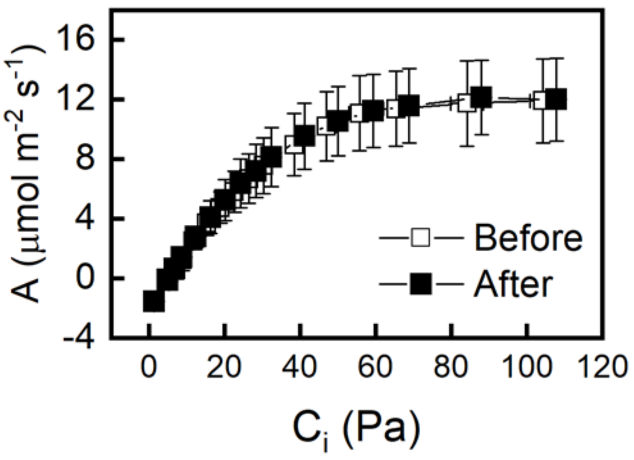

Supplementary Figure S2. A – C_i curves of WS taken before and after 4 hr incubation at a PFD of 500 µmol m_{-2} s^{-1} and C_a of 400 ppm. A – C_i curves were done at a PFD of 500 µmol m^{-2} s^{-1} n=5

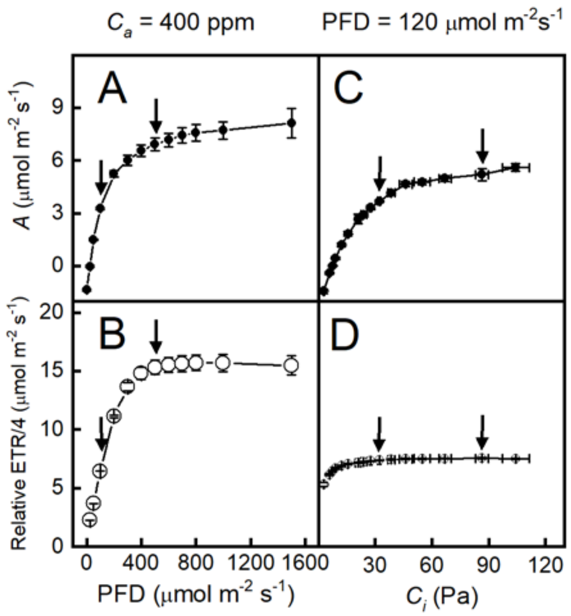

Supplementary Figure S3. Photosynthetic and relative electron transport (ETR) rates in Col-0 in varying light and CO_2 environments. Arrows indicate the PFD or average C_i values that correspond to the PFD or C_a values used in transcript experiments. ETR/4 is taken as the relative approximate rate of the Calvin-Benson cycle turnover. n=5

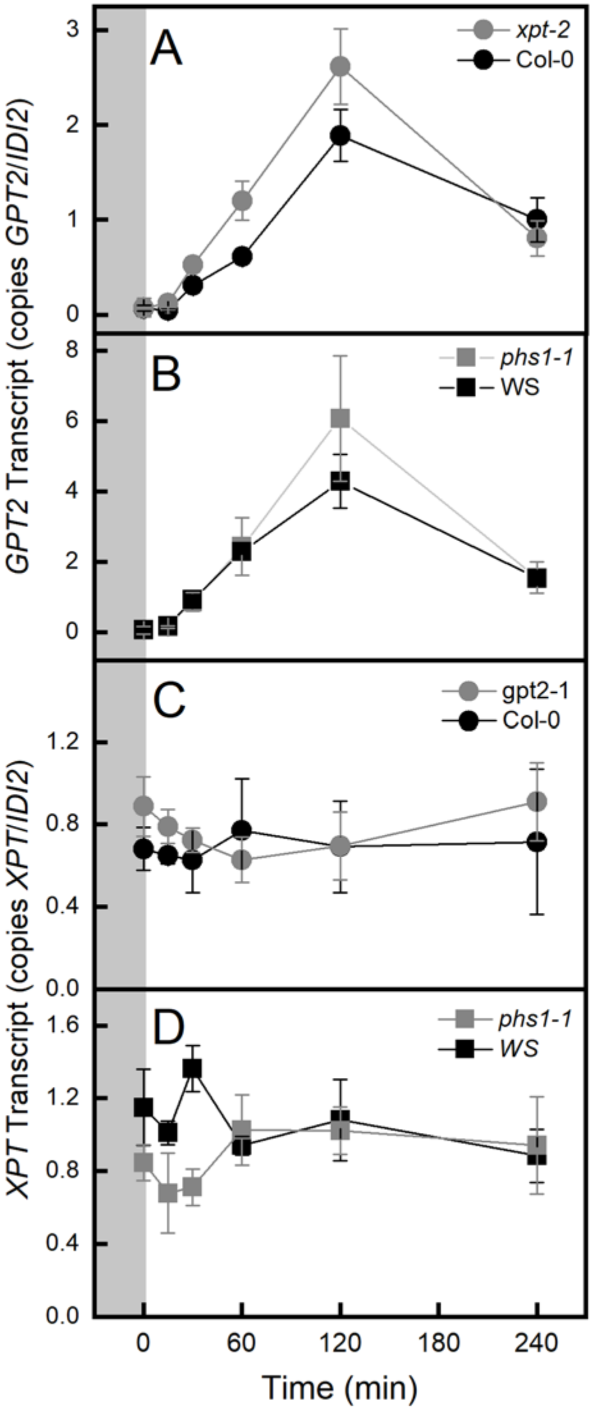

Supplementary Figure S4. Expression of *GPT2* and *XPT* in *xpt-2*, *gpt2-1,* and *phs1-1* mutants in response to a PFD of 500 μmol m^{-2} s^{-1} C_a of 400 ppm. The grey block indicates sampling in growth conditions, PFD 120 μmol m^{-2} s^{-1} and 400 ppm CO_2. n = 5

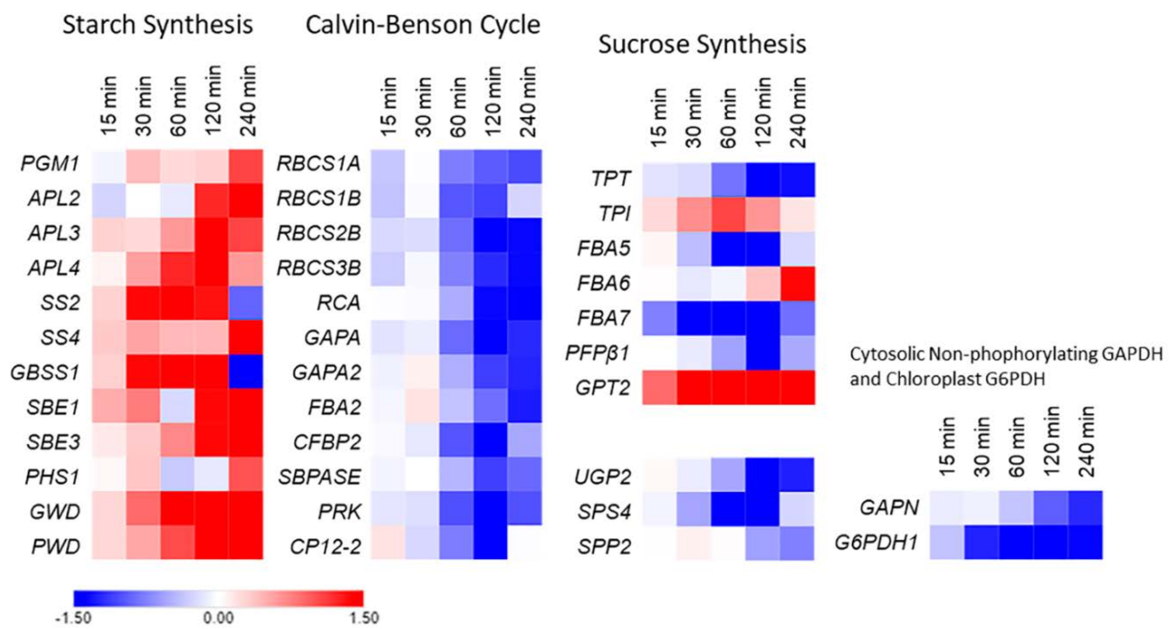

Supplementary Figure S5. Changes in transcription in WS in response to an increase in PFD from 120 to 500 µmol m^{-2} s^{-1}. Red boxes represent an increase in transcription and blue a decrease. Expression values shown had a log_2 RPKM fold change greater than1 and are on an absolute scale of -1.5 to 1.5 n=3

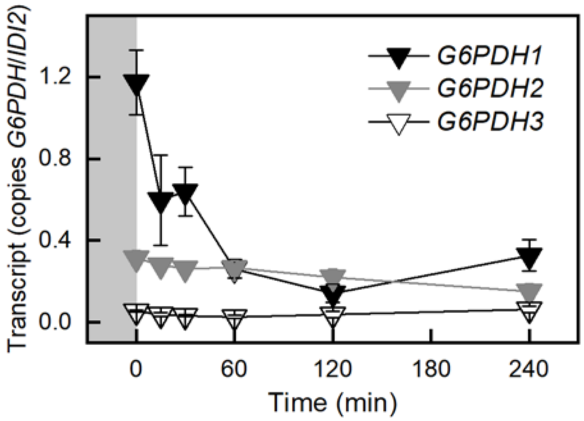

Supplementary Figure S6. Changes in transcription of the plastid *G6PDH*s in WS in response to a PFD of 500 µmol m^{-2} s^{-1} C_a 400 ppm. Grey block indicates sampling in growth conditions, PFD 120 µmol m^{-2} s^{-1} and 400 ppm CO_2. n=5

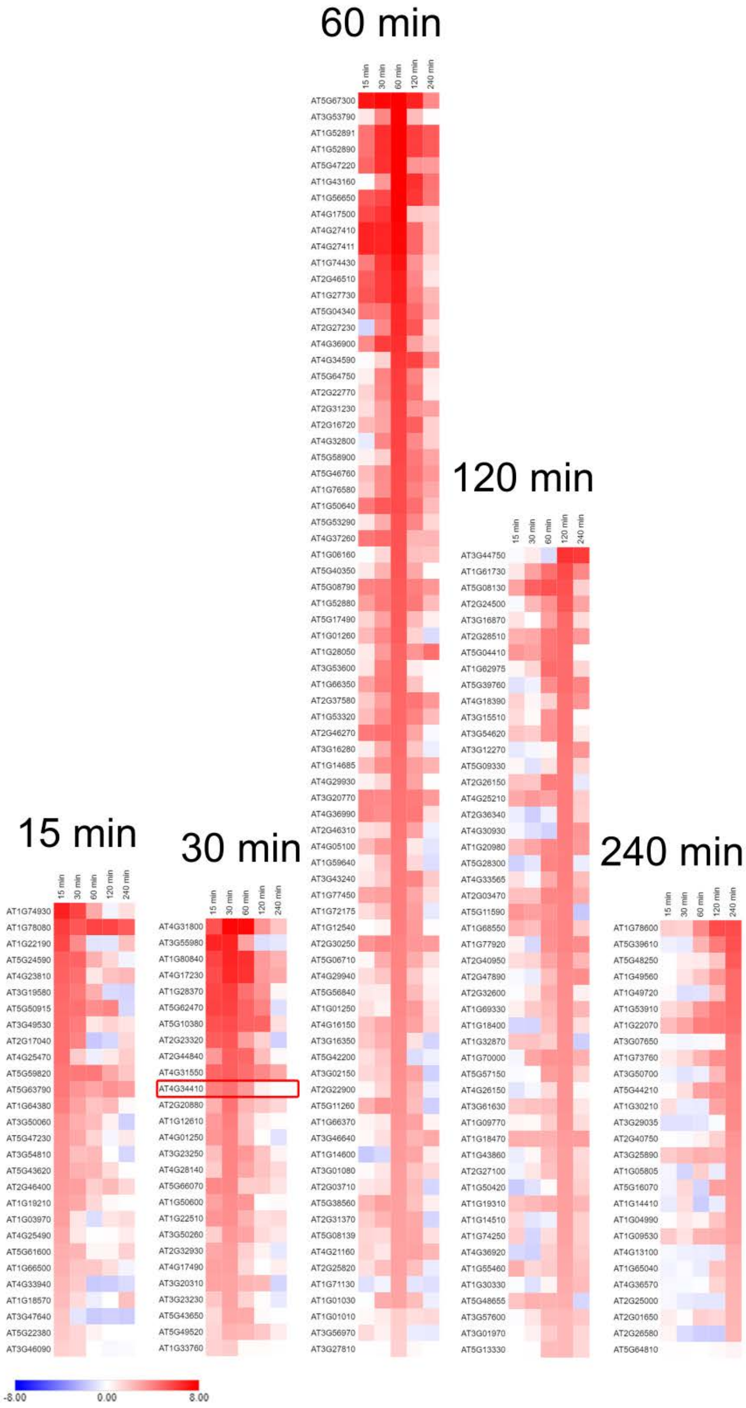

Supplementary Figure S7. Transcription factors in WS whose expression was increased in response to an increase in PFD from 120 to 500 µmol m^{-2} s^{-1}. Transcription factors are grouped according to the time they had the highest transcript abundance after transfer to high light. The red box is around the *RRTF1* transcription factor. Expression values used for heat map generation are the $\log_2$ ΔRPKM + 1 and are on an absolute scale of -8 to 8. n=3

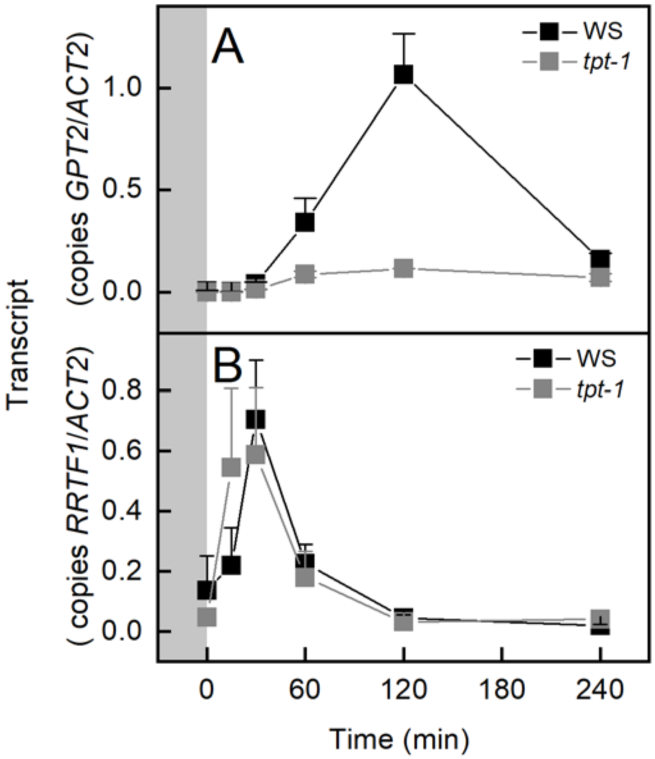

Supplementary Figure S8. Expression of *GPT2* and *RRTF1* in response to an increase in PFD from 120 to 500 µmol m^{-2} s^{-1} C_a 400 ppm in WS and the *tpt-1* mutant. Grey block indicates sampling in growth conditions, PFD 120 µmol m^{-2} s^{-1} and 400 ppm CO_2. n=5
