# Supplementary material for: Transcriptional Regulation of the Glucose-6-Phosphate/Phosphate Translocator 2 Is Related to Carbon Exchange Across the Chloroplast Envelope
Source: Front Plant Sci. 2019 Jun 27;10:827. doi: 10.3389/fpls.2019.00827 (PMC6609314; doi:10.3389/fpls.2019.00827)
Supplement: Supplementary file 2 [file Presentation_2.pdf]

Transcriptional regulation of the glucose-6-phosphate transporter 2

Supplemental Appendix S1. Specific Arabidopsis gene loci for specific biochemical pathways

Sean E. Weise[1,2], Tiffany Liu[3], Kevin L. Childs[3], Alyssa L. Preiser[1], Hailey M. Katulski[1], Christopher Perrin-Porzondek[1], Thomas D. Sharkey[1,2,4 *]

[1]MSU-DOE-Plant Research Laboratory, Michigan State University, East Lansing, MI USA
[2]Department of Biochemistry and Molecular Biology, Michigan State University, East Lansing, MI USA
[3]Department of Plant Biology, Michigan State University, East Lansing, MI USA
[4]Plant Resilience Institute, Michigan State University, East Lansing, MI USA

Calvin-Benson Cycle

Gene	Name	Gene Locus	Reference
Rubisco large subunit	RBCL	AtCg00490	Peltier et al., 2006
Rubisco small subunit chain 1A	RBCS1A	At1g67090	Krebbers et al., 1988
Rubisco small subunit chain 1B	RBCS1B	At5g38430	Krebbers et al., 1988
Rubisco small subunit chain 2B	RBCS2B	At5g38420	Krebbers et al., 1988
Rubisco small subunit chain 3B	RBCS3B	At5g38410	Krebbers et al., 1988
Rubisco activase	RCA	At2g39730	Werneke and Ogren, 1989
Phosphoglycerate kinase 1	PGK1	At3g12780	Rosa-Téllez et al., 2018
Phosphoglycerate kinase 2	PGK2	At1g56190	Rosa-Téllez et al., 2018
Glyceraldehyde-3-phosphate dehydrogenase A subunit	GAPA-1	At3g26650	Marri et al., 2005
Glyceraldehyde-3-phosphate dehydrogenase A-2 subunit	GAPA-2	At1g12900	Marri et al., 2005; Peltier et al., 2006
Glyceraldehyde-3-phosphate dehydrogenase B subunit	GAPB	At1g42970	Marri et al., 2005
Triosephosphate isomerase	TPI	At2g21170	Chen and Thelen, 2010
Fructose-1,6-bisphosphate aldolase 1	FBA1	At2g1330	Lu et al., 2012
Fructose-1,6-bisphosphate aldolase 2	FBA2	At4g38970	Lu et al., 2012
Fructose-1,6-bisphosphate aldolase 3	FBA3	At2g01140	Lu et al., 2012
Frucotse-1,6-bisphosphatase	CFBP1	At3g54050	Serrato et al., 2009a
Frucotse-1,6-bisphosphatase	CFBP2	At5g64380	Serrato et al., 2009b
Transketolase	TKL1	At3g60750	Rocha et al., 2014
Sedoheptulose-1,7-bisphosphatase	SBPASE	At3g55800	Willingham et al., 1994
Ribose-5-phosphate isomerase	EMB3119	At3g04790	Howles et al., 2006
Ribulose-phosphate-3-epimerase	RPE	At5g61410	Favery et al., 1998

Phosphoribulokinase	PRK	At1g32060	Marri et al., 2005
Chloroplast protein 12-1	CP12-1	At2g47400	Singh et al., 2008
Chloroplast protein 12-2	CP12-2	At3g62410	Singh et al., 2008

Chloroplast G6P Shunt

Gene	Name	Gene Locus	Reference
Glucose-6-phosphate dehydrogenase 1	G6PDH1	At5g35790	Wakao and Benning, 2005
Glucose-6-phosphate dehydrogenase 2	G6PDH2	At5g13110	Wakao and Benning, 2005
Glucose-6-phosphate dehydrogenase 3	G6PDH3	At1g24280	Wakao and Benning, 2005
6-Phosphogluconolactonase 3	6PGL3	At5g24400	Xiong et al., 2009
6-Phosphogluconate dehydrogenase 1	PGD1	At1g64190	Hölscher et al., 2016
6-Phosphogluconate dehydrogenase 2	PD2	At3g02360	Hölscher et al., 2016

Starch Synthesis

Gene	Name	Gene Locus	Reference
Phosphoglucoisomerase 1	PGI1	At4g24620	Yu et al., 2000
Phosphoglucomutase 1	PGM1	At5g51820	Egli et al., 2010
ADP glucose pyrophosphorylase large subunit 1	APL1	At5g19220	Crevillén et al., 2005
ADP glucose pyrophosphorylase large subunit 2	APL2	At1g27680	Crevillén et al., 2005
ADP glucose pyrophosphorylase large subunit 3	APL3	At4g39210	Crevillén et al., 2005
ADP glucose pyrophosphorylase large subunit 4	APL4	At2g21590	Crevillén et al., 2005
ADP glucose pyrophosphorylase small subunit 1	APS1	At5g48300	Hendriks et al., 2003
Starch synthase 1	SS1	At5g24300	Delvallé et al., 2005
Starch synthase 2	SS2	At3g01180	Zhang et al., 2008
Starch synthase 3	SS3	At1g11720	Zhang et al., 2005
Starch synthase 4	SS4	At4g18240	Roldán et al., 2007
Granule bound starch synthase1	GBSS1	At1g32900	Tenorio et al., 2003
Starch branching enzyme1	BE1	At3g20440	Wang et al., 2010
Starch branching enzyme2	BE2	At5g03650	Dumez et al., 2006
Starch branching enzyme 3	BE3	At2g36390	Dumez et al., 2006
Isoamylase 1	ISA1	At2g39930	Delatte et al., 2005
Isoamylase 2	ISA2	At1g03310	Delatte et al., 2005
Glucan water dikinase 1	GWD 1	At1g10760	Ritte et al., 2002
Phosphoglucan water dikinase	PWD	At5g26570	Kötting et al., 2005

Sucrose Synthesis

Gene	Name	Gene Locus	Reference
Triose phosphate transporter	TPT	At5g46110	Schneider et al., 2002
Triose phosphate isomerase	CYTOTPI	At3g55440	Shih, 1994
Fructose-1,6-bisphosphate aldolase 5	FBA5	At4g26530	Lu et al., 2012
Fructose-1,6-bisphosphate aldolase 6	FBA6	At2g36460	Lu et al., 2012
Fructose-1,6-bisphosphate aldolase 7	FBA7	At4g26520	Lu et al., 2012
Fructose-1,6-bisphosphatase	CYFBP	At1g43670	García-Díaz et al., 2015
Pyrophosphate dependent fructose-6-phosphate 1-phosphotransferase α1 subunit	PFPα1	At1g20950	Lim et al., 2009
Pyrophosphate dependent fructose-6-phosphate 1-phosphotransferase α2 subunit	PFPα2	At1g76550	Lim et al., 2009
Pyrophosphate dependent fructose-6-phosphate 1-phosphotransferase β1 subunit	PFPβ1	At1g12000	Lim et al., 2009
Pyrophosphate dependent fructose-6-phosphate 1-phosphotransferase β2 subunit	PFPβ2	At4g04040	Lim et al., 2009
Phosphoglucoisomerase 2	PGI2	At5g42740	Kunz et al., 2014
Phosphoglucomutase 2	PGM2	At1g70730	Egli et al., 2010
Phosphoglucomutase 3	PGM3	At1g23190	Egli et al., 2010
UDP-glucose pyrophosphorylase 1	UGP1	At3g03250	Meng et al., 2008
UDP-glucose pyrophosphorylase 2	UGP2	At5g17310	Meng et al., 2008
Sucrose phosphate synthase A1	SPSA1	At5g20280	Volkert et al., 2014
Sucrose phosphate synthase A2	SPSA2	AT5g11110	Volkert et al., 2014
Sucrose phosphate synthase B	SPSB	At1g04920	Volkert et al., 2014
Sucrose phosphate synthase C	SPSC	At4g10120	Volkert et al., 2014
Sucrose phosphate phosphatase 2	SPP2	At2g35840	Albi et al., 2016
Sucrose phosphate phosphatase 3A	SPP3A	At3g54270	Albi et al., 2016
Sucrose phosphate phosphatase 3B	SPP3B	At3g52340	Albi et al., 2016

Cytosolic Glycolysis

Gene	Name	Gene Locus	Reference
Glyceraldehyde-3-phosphate dehydrogenase C1	GAPC1	At3g04120	Rius et al., 2008
Glyceraldehyde-3-phosphate dehydrogenase C2	GAPC2	At1g13440	Guo et al., 2014
Phosphoglycerate kinase 3	PGK3	At1g79550	Rosa-Téllez et al., 2018
Non-phosphorylating glyceraldehyde-3-phosphate dehydrogenase	GAPN	At2g24270	Rius et al., 2006
Phosphoglycerate mutase	iPGAM1	At1g09780	Zhao and Assmann, 2011
Phosphoglycerate mutase	iPGAM2	At3g08590	Zhao and Assmann, 2011
Enolase	ENO2	At2g36530	Eremina et al., 2015
Predicted cytosolic pyruvate kinase	PK1	At5g08570	Yanagisawa et al., 2004
Predicted cytosolic pyruvate kinase	PK2	At5g63680	Yanagisawa et al., 2004
Predicted cytosolic pyruvate kinase		At3g52990	Andre et al., 2007
Predicted cytosolic pyruvate kinase		At2g36580	Andre et al., 2007
Predicted cytosolic pyruvate kinase		At3g55650	Andre et al., 2007
Predicted cytosolic pyruvate kinase		At5g56350	Andre et al., 2007
Predicted cytosolic pyruvate kinase		At4g26390	Andre et al., 2007
Predicted cytosolic pyruvate kinase		At3g04050	Andre et al., 2007
Predicted cytosolic pyruvate kinase		At3g25960	Andre et al., 2007
Predicted cytosolic pyruvate kinase		At3g55810	Andre et al., 2007

Albi, T., Ruiz, M. T., de los Reyes, P., Valverde, F., and Romero, J. M. (2016). Characterization of the sucrose phosphate phosphatase (SPP) isoforms from *Arabidopsis thaliana* and role of the S6PPc domain in dimerization. *PLoS ONE*. 11, e0166308. doi: 10.1371/journal.pone.0166308

Andre, C., Froehlich, J. E., Moll, M. R., and Benning, C. (2007). A heteromeric plastidic pyruvate kinase complex involved in seed oil biosynthesis in *Arabidopsis*. *Plant Cell*. 19, 2006-2022. doi: 10.1105/tpc.106.048629

Chen, M., and Thelen, J. J. (2010). The plastid isoform of triose phosphate isomerase is required for the postgerminative transition from heterotrophic to autotrophic growth in *Arabidopsis*. *Plant Cell*. 22, 77-90. doi: 10.1105/tpc.109.071837

Crevillén, P., Ventriglia, T., Pinto, F., Orea, A., Mérida, Á., and Romero, J. M. (2005). Differential pattern of expression and sugar regulation of *Arabidopsis thaliana* ADP-glucose pyrophosphorylase-encoding genes. *J. Biol. Chem.* 280, 8143-8149. doi: 10.1074/jbc.M411713200

Delatte, T., Trevisan, M., Parker, M. L., and Zeeman, S. C. (2005). *Arabidopsis* mutants Atisa1 and Atisa2 have identical phenotypes and lack the same multimeric isoamylase, which influences the branch point distribution of amylopectin during starch synthesis. *Plant J.* 41, 815-830. doi: 10.1111/j.1365-313X.2005.02348.x

Delvallé, D., Dumez, S., Wattebled, F., Roldán, I., Planchot, V., Berbezy, P., et al. (2005). Soluble starch synthase I: a major determinant for the synthesis of amylopectin in

Arabidopsis thaliana leaves. *Plant J.* 43, 398-412. doi: doi:10.1111/j.1365-313X.2005.02462.x
Dumez, S., Wattebled, F., Dauvillee, D., Delvalle, D., Planchot, V., Ball, S. G., et al. (2006). Mutants of *Arabidopsis* lacking starch branching enzyme II substitute plastidial starch synthesis by cytoplasmic maltose accumulation. *Plant Cell.* 18, 2694-2709. doi: 10.1105/tpc.105.037671
Egli, B., Kolling, K., Kohler, C., Zeeman, S. C., and Streb, S. (2010). Loss of cytosolic phosphoglucomutase compromises gametophyte development in Arabidopsis. *Plant Physiol.* 154, 1659-1671. doi: 10.1104/pp.110.165027
Eremina, M., Rozhon, W., Yang, S., and Poppenberger, B. (2015). ENO2 activity is required for the development and reproductive success of plants, and is feedback-repressed by AtMBP-1. *Plant J.* 81, 895-906. doi: doi:10.1111/tpj.12775
Favery, B., Lecomte, P., Gil, N., Bechtold, N., Bouchez, D., Dalmasso, A., et al. (1998). RPE, a plant gene involved in early developmental steps of nematode feeding cells. *The EMBO Journal.* 17, 6799-6811. doi: 10.1093/emboj/17.23.6799
García-Díaz, Á., Serrato, A. J., Rojas-González, J. A., Sandalio, L. M., Romero-Puertas, M. C., Soto-Súarez, M., et al. (2015). Disruption of both chloroplastic and cytosolic FBPase genes results in a dwarf phenotype and important starch and metabolite changes in Arabidopsis thaliana. *J. Exp. Bot.* 66, 2673-2689. doi: 10.1093/jxb/erv062
Guo, L., Ma, F., Wei, F., Fanella, B., Allen, D. K., and Wang, X. (2014). Cytosolic phosphorylating glyceraldehyde-3-phosphate dehydrogenases affect *Arabidopsis* cellular metabolism and promote seed oil accumulation. *Plant Cell.* 26, 3023-3035. doi: 10.1105/tpc.114.126946
Hendriks, J. H. M., Kolbe, A., Gibon, Y., Stitt, M., and Geigenberger, P. (2003). ADP-glucose pyrophosphorylase is activated by posttranslational redox-modification in response to light and to sugars in leaves of Arabidopsis and other plant species. *Plant Physiol.* 133, 838-849. doi:
Hölscher, C., Lutterbey, M.-C., Lansing, H., Meyer, T., Fischer, K., and von Schaewen, A. (2016). Defects in peroxisomal 6-phosphogluconate dehydrogenase isoform *PGD2* Prevent Gametophytic Interaction in *Arabidopsis thaliana*. *Plant Physiol.* 171, 192-205. doi: 10.1104/pp.15.01301
Howles, P. A., Birch, R. J., Collings, D. A., Gebbie, L. K., Hurley, U. A., Hocart, C. H., et al. (2006). A mutation in an *Arabidopsis* ribose 5-phosphate isomerase reduces cellulose synthesis and is rescued by exogenous uridine. *Plant J.* 48, 606-618. doi: 10.1111/j.1365-313X.2006.02902.x
Kötting, O., Pusch, K., Tiessen, A., Geigenberger, P., Steup, M., and Ritte, G. (2005). Identification of a novel enzyme required for starch metabolism in Arabidopsis leaves. The phosphoglucan, water dikinase. *Plant Physiol.* 137, 242-252. doi:
Krebbers, E., Seurinck, J., Herdies, L., Cashmore, A. R., and Timko, M. P. (1988). Four genes in two diverged subfamilies encode the ribulose-1,5-bisphosphate carboxylase small subunit polypeptides of *Arabidopsis thaliana*. *Plant Mol. Biol.* 11, 745-759. doi: 10.1007/bf00019515
Kunz, H.-H., Zamani-Nour, S., Häusler, R. E., Ludewig, K., Schroeder, J. I., Malinova, I., et al. (2014). Loss of cytosolic phosphoglucose isomerase affects carbohydrate metabolism in leaves and is essential for fertility of *Arabidopsis*. *Plant Physiol.* 166, 753-765. doi: 10.1104/pp.114.241091

Lim, H., Cho, M.-H., Jeon, J.-S., Bhoo, S. H., Kwon, Y.-K., and Hahn, T.-R. (2009). Altered expression of pyrophosphate: Fructose-6-phosphate 1-phosphotransferase affects the growth of transgenic Arabidopsis plants. *Molecules and Cells*. 27, 641-649. doi: 10.1007/s10059-009-0085-0

Lu, W., Tang, X. L., Huo, Y. Q., Xu, R., Qi, S. D., Huang, J. G., et al. (2012). Identification and characterization of fructose 1,6-bisphosphate aldolase genes in *Arabidopsis* reveal a gene family with diverse responses to abiotic stresses. *Gene*. 503, 65-74. doi: 10.1016/j.gene.2012.04.042

Marri, L., Sparla, F., Pupillo, P., and Trost, P. (2005). Co-ordinated gene expression of photosynthetic glyceraldehyde-3-phosphate dehydrogenase, phosphoribulokinase, and CP12 in Arabidopsis thaliana. *J. Exp. Bot.* 56, 73-80. doi:

Meng, M., Wilczynska, M., and Kleczkowski, L. A. (2008). Molecular and kinetic characterization of two UDP-glucose pyrophosphorylases, products of distinct genes, from *Arabidopsis*. *Biochimica et Biophysica Acta (BBA) - Proteins and Proteomics*. 1784, 967-972. doi: 10.1016/j.bbapap.2008.02.021

Peltier, J.-B., Cai, Y., Sun, Q., Zabrouskov, V., Giacomelli, L., Rudella, A., et al. (2006). The oligomeric stromal proteome of *Arabidopsis thaliana* chloroplasts. *Mol. Cell. Proteomics*. 5, 114-133. doi: 10.1074/mcp.M500180-MCP200

Ritte, G., Lloyd, J. R., Eckermann, N., Rottmann, A., Kossmann, J., and Steup, M. (2002). The starch-related R1 protein is an α-glucan, water dikinase. *Proc. Natl Acad. Sci. USA*. 99, 7166-7171. doi: 10.1073/pnas.062053099

Rius, S. P., Casati, P., Iglesias, A. A., and Gomez-Casati, D. F. (2006). Characterization of an *Arabidopsis thaliana* mutant lacking a cytosolic non-phosphorylating glyceraldehyde-3-phosphate dehydrogenase. *Plant Mol. Biol.* 61, 945-957. doi: 10.1007/s11103-006-0060-5

Rius, S. P., Casati, P., Iglesias, A. A., and Gomez-Casati, D. F. (2008). Characterization of *Arabidopsis* lines deficient in GAPC-1, a cytosolic NAD-dependent glyceraldehyde-3-phosphate dehydrogenase. *Plant Physiol.* 148, 1655-1667. doi: 10.1104/pp.108.128769

Rocha, A. G., Mehlmer, N., Stael, S., Mair, A., Parvin, N., Chigri, F., et al. (2014). Phosphorylation of *Arabidopsis* transketolase at Ser^{428} provides a potential paradigm for the metabolic control of chloroplast carbon metabolism. *Biochem. J.* 458, 313-322. doi: 10.1042/bj20130631

Roldán, I., Wattebled, F., Mercedes Lucas, M., Delvallé, D., Planchot, V., Jiménez, S., et al. (2007). The phenotype of soluble starch synthase IV defective mutants of *Arabidopsis thaliana* suggests a novel function of elongation enzymes in the control of starch granule formation. *Plant J.* 49, 492-504. doi: doi:10.1111/j.1365-313X.2006.02968.x

Rosa-Téllez, S., Anoman, A. D., Flores-Tornero, M., Toujani, W., Alseek, S., Fernie, A. R., et al. (2018). Phosphoglycerate kinases are co-regulated to adjust metabolism and to optimize growth. *Plant Physiol.* 176, 1182-1198. doi: 10.1104/pp.17.01227

Schneider, A., Häusler, R. E., Kolukisaoglu, Ü., Kunze, R., van Der Graaff, E., Schwacke, R., et al. (2002). An *Arabidopsis thaliana* knock-out mutant of the chloroplast triose phosphate/phosphate translocator is severely compromised only when starch synthesis, but not starch mobilisation is abolished. *Plant J.* 32, 685-699. doi: 10.1046/j.1365-313X.2002.01460.x

Serrato, A. J., Barajas-Lopez, J. D., Chueca, A., and Sahrawy, M. (2009a). Changing sugar partitioning in FBPase-manipulated plants. *J. Exp. Bot.* 60, 2923-2931. doi: 10.1093/jxb/erp066

Serrato, A. J., Yubero-Serrano, E. M., Sandalio, L. M., Munoz-Blanco, J., Chueca, A., Caballero, J. L., et al. (2009b). cpFBPaseII, a novel redox-independent chloroplastic isoform of fructose-1,6-bisphosphatase. *Plant Cell Environ.* 32, 811-827. doi: 10.1111/j.1365-3040.2009.01960.x

Shih, M. C. (1994). Cloning and sequencing of a cDNA clone encoding the cytosolic triose-phosphate isomerase from *Arabidopsis thaliana*. *Plant Physiol.* 104, 1103-1104. doi: 10.1104/pp.104.3.1103

Singh, P., Kaloudas, D., and Raines, C. A. (2008). Expression analysis of the *Arabidopsis* CP12 gene family suggests novel roles for these proteins in roots and floral tissues. *J. Exp. Bot.* 59, 3975-3985. doi: 10.1093/jxb/ern236

Tenorio, G., Orea, A., Romero, J. M., and Mérida, Á. (2003). Oscillation of mRNA level and activity of granule-bound starch synthase I in *Arabidopsis* leaves during the day/night cycle. *Plant Mol. Biol.* 51, 949-958. doi: 10.1023/a:1023053420632

Volkert, K., Debast, S., Voll, L. M., Voll, H., Schießl, I., Hofmann, J., et al. (2014). Loss of the two major leaf isoforms of sucrose-phosphate synthase in *Arabidopsis thaliana* limits sucrose synthesis and nocturnal starch degradation but does not alter carbon partitioning during photosynthesis. *J. Exp. Bot.* 65, 5217-5229. doi: 10.1093/jxb/eru282

Wakao, S., and Benning, C. (2005). Genome-wide analysis of glucose-6-phosphate dehydrogenases in Arabidopsis. *Plant J.* 41, 243-256. doi: 10.1111/j.1365-313X.2004.02293.x

Wang, X., Xue, L., Sun, J., and Zuo, J. (2010). The *Arabidopsis* BE1 gene, encoding a putative glycoside hydrolase localized in plastids, plays crucial roles during embryogenesis and carbohydrate metabolism. *Journal of Integrative Plant Biology*. 52, 273-288. doi: 10.1111/j.1744-7909.2010.00930.x

Werneke, J. M., and Ogren, W. L. (1989). Structure of an *Arabidopsis thaliana* cDNA encoding rubisco activase. *Nucleic Acids Res.* 17, 2871-2871. doi: 10.1093/nar/17.7.2871

Willingham, N. M., Lloyd, J. C., and Raines, C. A. (1994). Molecular-cloning of the *Arabidopsis thaliana* sedoheptulose-1,7-bisphosphatase gene and expression studies in wheat and *Arabidopsis thaliana*. *Plant Mol. Biol.* 26, 1191-1200. doi: 10.1007/bf00040699

Xiong, Y. Q., DeFraia, C., Williams, D., Zhang, X. D., and Mou, Z. L. (2009). Characterization of *Arabidopsis* 6-phosphogluconolactonase T-DNA insertion mutants reveals an essential role for the oxidative section of the plastidic pentose phosphate pathway in plant growth and development. *Plant and Cell Physiology*. 50, 1277-1291. doi: 10.1093/pcp/pcp070

Yanagisawa, S., Akiyama, A., Kisaka, H., Uchimiya, H., and Miwa, T. (2004). Metabolic engineering with Dof1 transcription factor in plants: Improved nitrogen assimilation and growth under low-nitrogen conditions. *Proc. Natl Acad. Sci. USA*. 101, 7833-7838. doi: 10.1073/pnas.0402267101

Yu, T.-S., Lue, W.-L., Wang, S.-M., and Chen, J. (2000). Mutation of *Arabidopsis* plastid phosphoglucose isomerase affects leaf starch synthesis and floral initiation. *Plant Physiol.* 123, 319-326. doi: 10.1104/pp.123.1.319

Zhang, X., Szydlowski, N., Delvallé, D., D'Hulst, C., James, M. G., and Myers, A. M. (2008). Overlapping functions of the starch synthases SSII and SSIII in amylopectin biosynthesis in *Arabidopsis*. *BMC Plant Biol.* 8, 96. doi: 10.1186/1471-2229-8-96

Zhang, X. L., Myers, A. M., and James, M. G. (2005). Mutations affecting starch synthase III in *Arabidopsis* alter leaf starch structure and increase the rate of starch synthesis. *Plant Physiol.* 138, 663-674. doi: 10.1104/pp.105.060319

Zhao, Z., and Assmann, S. M. (2011). The glycolytic enzyme, phosphoglycerate mutase, has critical roles in stomatal movement, vegetative growth, and pollen production in *Arabidopsis thaliana*. *J. Exp. Bot.* 62, 5179-5189. doi: 10.1093/jxb/err223
